# Stress-testing pelvic autosegmentation algorithms using anatomical edge cases

**DOI:** 10.1016/j.phro.2023.100413

**Published:** 2023-01-16

**Authors:** Aasheesh Kanwar, Brandon Merz, Cheryl Claunch, Shushan Rana, Arthur Hung, Reid F. Thompson

**Affiliations:** a Department of Radiation Medicine, Oregon Health and Sciences University, Portland, OR, United States; b Department of Radiation Oncology, Baylor College of Medicine, Houston, TX, United States; c PeaceHealth Medical Group – PeaceHealth Southwest Radiation Oncology, Vancouver, Washington, United States; d Division of Hospital and Specialty Medicine, VA Portland Healthcare System, Portland, OR, United States

**Keywords:** Deep learning, Autosegmentation, Edge case, Prostate cancer, Anatomical variability

## Abstract

Commercial autosegmentation has entered clinical use, however real-world performance may suffer in certain cases. We aimed to assess the influence of anatomic variants on performance. We identified 112 prostate cancer patients with anatomic variations (edge cases). Pelvic anatomy was autosegmented using three commercial tools. To evaluate performance, Dice similarity coefficients, and mean surface and 95% Hausdorff distances were calculated versus clinician-delineated references. Deep learning autosegmentation outperformed atlas-based and model-based methods. However, edge case performance was lower versus the normal cohort (0.12 mean DSC reduction). Anatomic variation presents challenges to commercial autosegmentation.

## Highlights

• Anatomical edge cases can degrade pelvic autosegmentation algorithm performance.
• Autosegmentation performance is case-specific and highly variable.
• Autosegmentation performs worse on average for cases with anatomical abnormalities.

## 1 Introduction

Artificial intelligence (AI) is emerging as a powerful transformative technology, with numerous applications in the radiation oncology clinic. In particular, autosegmentation algorithms, which automatically delineate structures of interest from imaging data, have demonstrated compelling accuracy across numerous sites [1], [2], [3]. Autosegmentation algorithms have also demonstrated the potential to improve clinical efficiency [4], [5], to standardize a high level of accuracy of segmented volumes across providers [6], and to enable more complex tasks such as automated treatment planning [7]. Accordingly, a wide variety of commercial and home-grown autosegmentation tools are being rapidly adopted and deployed in the clinic [8], [9].

However, as autosegmentation algorithms proliferate in both their existence and use in the clinic, the potential for real world harm increases [10]. AI algorithms are frequently subject to deep, implicit biases, potentially threatening their generalized use in more diverse settings [11], [12]. Identifying such issues prospectively, and on a per-patient basis, remains a particularly daunting challenge. Edge cases – situations that occur at extreme values [“edges”] of an expected distribution and which may present scenarios not encountered during AI training – are a principal source of this issue, and are an inherent result of the real-world heterogeneity across individuals and circumstances [13]. However, the radiation oncology literature is largely devoid of edge case assessments of autosegmentation tools.

In this study, we sought to evaluate the influence of edge cases (consisting in this case of eight different classes of uncommon anatomical variation) on the performance of three distinct commercial autosegmentation algorithms.

## 2 Methods and materials

### 2.1 Clinical cohort classification

All work and other study activities were conducted under institutional IRB approval.

We identified a cohort of 950 consecutive prostate cancer cases receiving definitive external beam radiotherapy at a single institution between 2011 and 2019. Each case was screened by a trained physician for the presence of any one of the following eight classes of anatomical variants: 1) prostate hypertrophy (i.e. median lobe hypertrophy, overall glandular hypertrophy), 2) elongated – or so-called “droopy” – seminal vesicles, 3) hip arthroplasty, 4) prostate surface irregularity or extracapsular extension, 5) prostate-intrinsic metal content (i.e. prostatic calcifications, fiducials, or low dose rate brachytherapy seeds), 6) in-dwelling Foley catheter, 7) SpaceOAR™ hydrogel, or 8) other notable variation per clinician discretion (i.e. in-field bowel, narrow rectum, morbid obesity [BMI ≥ 50]). Each identified edge case (n = 112) was annotated as being among any of these eight classes, and it was possible for an edge case to have more than one flagged anatomical variant. A separate cohort of “normal” cases (n = 19) was randomly selected from individuals without any of the eight classes of anatomical variants above. The cohort was summarized in Supplementary Table 1, and detailed individually in Supplementary Table 2.

### 2.2 Structure segmentation

Target and organ at risk (OAR) contours (prostate, rectum, bladder, and bilateral femoral heads) were manually delineated on simulation CT scans by a single radiation oncologist, paying reference to co-registered MRI images where available, and clinically approved and used for treatment planning following peer review. Manual contours were generated according to institutional standards derived from established consensus protocols. Where relevant, a research-specific rectum structure was extended from the clinically-approved structure to include the full length of rectum outside the delineated PTV. Three distinct autosegmentation tools were locally installed and run on hardware with a 10-core Xeon processor, 64 GB RAM, and 16 GB GPU implemented: 1) multi-subject atlas-based autosegmentation (AB) via intensity-based free-form deformable registration available from MIM Software Inc. (using the off-the-shelf high risk prostate atlas version 2.014, 2016 package, without any customization), 2) model-based segmentation (MB) available from RaySearch Laboratories (operating as a black-box without the use of structure ‘hint’ tools), and 3) a U-Net architecture [14] deep-learning segmentation (DL) model available from RaySearch Laboratories version 9B (v. 2.3.0) [15], also operating as a black-box. Note that the MB method is proprietary and employs statistical shape models as ready-to-use groups of structures, with parameters for these models specified internally by RaySearch. We refer the interested reader to a more nuanced discussion of the distinctions between these different autosegmentation approaches [16], [17].

All imaging and manually-delineated structure data, along with edge case labels and basic demographic data, have been deposited on the Cancer Imaging Archive (TCIA; https://www.cancerimagingarchive.net/) and are available for public access at https://doi.org/10.7937/Fqstf-st65.

### 2.3 Structure comparison

DICOM-RT structure set (RTSTRUCT) data was exported from Eclipse, and subsequently imported for analysis using the RadOnc package (v.1.1.8) [18] and R (v.4.0.3). For each structure type and autosegmentation approach, Sørensen-Dice similarity coefficients (DSC), mean surface distances (MSD), and 95 % Hausdorff distances (HD) were calculated for autosegmented structures compared to corresponding manually-delineated structures. Structure comparisons were detailed per individual in Supplementary Table 3–5.

## 3 Results

We identified 112 edge cases (11.8 %) that harbored one or more of eight distinct anatomical variants, with prostatic hypertrophy (5.5 %) being the single most common class of anatomical edge case (Supplementary Table 1). While the vast majority of identified edge cases contained a single class of anatomic variation, we identified a subset of the cohort (15.2 %) possessing two or more different classes of anatomic variation.

**Fig. 1:**
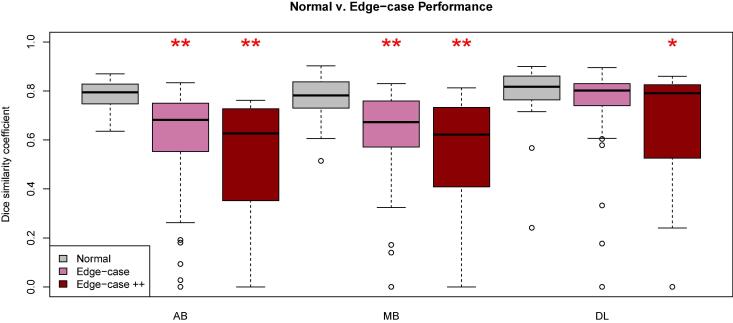
Overall performance of autosegmentation tools on normal and edge case cohorts. The distribution of Dice similarity coefficients (y-axis) is shown here as box plots for each of three cohorts of individuals (“Normal” shown in gray, “Edge-case” with a single anatomic variant shown in pink, and “Edge-case ++” with two or more simultaneous anatomic variants shown in dark red), where each datapoint is an average across all structures for that individual. Performance is reported for each of three autosegmentation tools: atlas-based autosegmentation [AB], model-based segmentation [MB], and deep-learning based segmentation [DL]). Statistically significant differences between normal and edge case performance are denoted by asterisks, where (*) and (**) represent p < 0.05 and p < 0.001, respectively (Wilcoxon Rank-Sum test). (For interpretation of the references to colour in this figure legend, the reader is referred to the web version of this article.)

Averaging across all structures, we noted no differences in autosegmentation performance between AB, MB, and DL in the normal cohort, with mean DSC [19] of 0.77, 0.76, and 0.78, respectively. However, AB and MB autosegmentation performance were significantly worse overall for anatomical edge cases with either a single abnormality (p < 0.001) or multiple classes of anatomic variation (p < 0.0001) compared to normal (Fig. 1, Supplementary Fig. 1). Overall performance of the DL algorithm was significantly worse among edge cases with multiple abnormalities compared to single abnormalities (p = 0.03) or the normal cohort (p = 0.04, with an average decrement of 0.12 DSC units) (Fig. 1).

In keeping with a known limitation of the DL algorithm, performance was especially poor across all structures in the presence of hip prostheses (Supplementary Figs. 2–4). Whereas, the presence of a Foley catheter appeared to primarily degrade performance for bladder segmentation (median DSC 0.95 → 0.72; p = 0.02) but not for rectum or femoral heads, while other anatomic variants (such as a narrow rectum or the presence of in-field bowel) significantly degraded performance for rectal segmentation (median DSC 0.63 → 0.37; p = 0.01). Presence of a SpaceOAR did not appear to degrade prostate segmentation in the majority of cases, but demonstrated wide variability in performance between cases across all structures. The performance across different classes of anatomical edge cases was distinct between algorithms (Supplementary Figs. 5 and 6). Note that autosegmentation performance was generally superior for bladder and femoral head structures compared with prostate or rectum (Supplementary Fig. 7).

**Fig. 2:**
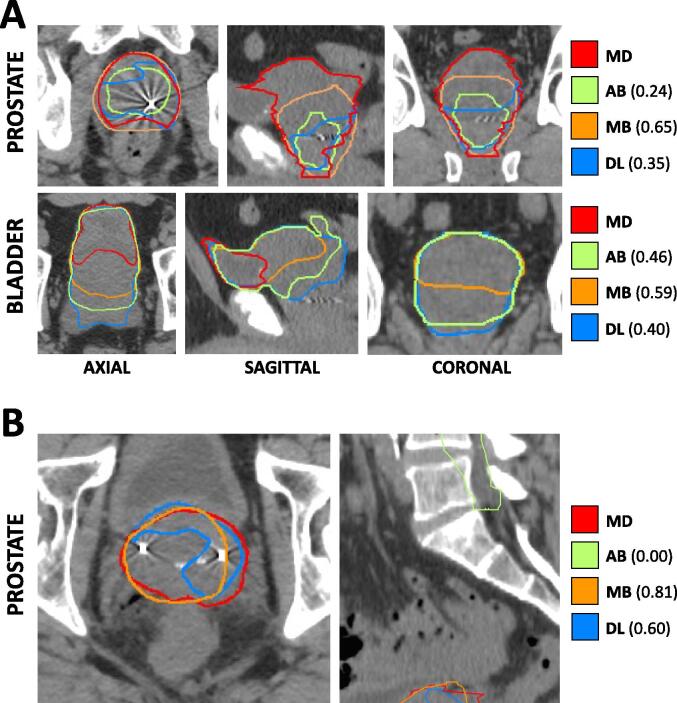
Cross-sectional CT-based anatomy and autosegmentation performance for representative edge cases. A) Hypertrophic prostate edge case. Each panel depicts a focused excerpt from a single CT scan, centered about two different structures (prostate, bladder) in three different planes (axial, sagittal, coronal). Clinician-delineated “ground truth” contours (MD) for each structure are shown in red, while atlas-based (AB), model-based (MB), and deep-learning based (DL) autosegmented contours are depicted in green, orange, and blue, respectively. Numerical values represent DSC for the corresponding autosegmented volumes compared to MD volumes. B) So-called “droopy” seminal vesicles edge case. Each panel depicts a focused excerpt from a single CT scan, centered about the prostate in two different planes (axial, sagittal). All colors and labeling are as in Panel A). (For interpretation of the references to colour in this figure legend, the reader is referred to the web version of this article.)

Interestingly, algorithm performance among individuals varied widely, both among edge cases within a single class of anatomic variation and even within the normal cohort (Supplementary Figs. 2–6, Supplementary Tables 3–5). For example, while prostatic hypertrophy as a broad cohort of anatomic variants performed reasonably well compared to the normal cohort, certain individual cases demonstrated particularly poor segmentation (Fig. 2A). The same phenomenon applied to other classes of anatomic variants, including so-called “droopy” seminal vesicles, where individual cases were particularly poor performers (Fig. 2B). The most significant outlier observed in the normal cohort had a short CT scan length, which may have deleteriously affected autosegmentation performance.

## 4 Discussion

This study assessed the influence of significant anatomic variants (edge cases) on the real-world performance of three commercial pelvic autosegmentation algorithms. Performance was compared with a cohort of normal cases without such anatomic variants.

While multiple studies have demonstrated good performance and clinical utility of autosegmentation tools [9], [20], we found that commercially-available autosegmentation algorithms differ substantially in performance and reliability. Our work demonstrated improved robustness of a deep learning algorithm over either an atlas or model-based approach, and this finding is in keeping with the performance improvements observed among various deep learning approaches in the literature [17], [21], [22]. Nonetheless, we found that anatomical edge cases pose distinct challenges for autosegmentation tools of at least three different types. This finding is directly in keeping with the fundamental caveats of machine and deep learning approaches that arise from mismatches between training and operational datasets [23]. We also described significant variety in performance for different structures among different edge cases, which may reflect structure-specific influences of different edge cases as well as innate discrepancies between the level of difficulty segmenting certain structures with higher or lower contrast soft-tissue boundaries [16]. To our knowledge, the evaluation of radiation oncology specific models using such edge cases represents a novel contribution to the field.

We note several limitations to this work. While we aimed for consistency by leveraging manually-derived contours from a single clinician at a single institution using images generated by a single CT scanner, we did not assess inter-observer variability or practice-level variation in contour delineation within or between institutions, nor technical variation in image quality or content associated with different CT scanning devices or parameters (e.g. scan length). Moreover, we did not assess the performance of many other available or emerging autosegmentation tools; while we hypothesize that the phenomena observed here apply generally across algorithms and anatomical sites, we have not demonstrated that explicitly in this work. We did not assess the potential dosimetric or downstream impacts of autosegmention among edge cases, nor the real-world implementation or clinical workflow incorporation of autosegmentation including time and effort savings, clinical acceptability, or risks of error propagation. Accordingly, it remains unclear whether statistically significant differences in performance translate to clinical significance. We note that our normal cohort was modestly sized and harbored its own outliers in algorithm performance. We also note that we were statistically underpowered to detect differences from normal performance in certain categories of edge cases with fewer examples (Supplementary Table 1). Finally, we were unable to investigate the inner workings of autosegmentation algorithms to better ascertain why performance varied so problematically for certain edge cases but not others, even within the same class of anatomical variants. Future work addressing these various limitations is certainly warranted.

As autosegmention is more widely adopted in the clinic, we anticipate that outliers may pose an ongoing need for identification and correction to ensure quality of care. However, while autosegmentation algorithm output may be readily apparent, numerous other classes of algorithms such as for outcome prediction, could prove challenging to interpret and therefore difficult to assess the robustness to anatomical or other edge cases. Our work suggests the potential importance of stress-testing existing algorithms (as well as those in-development) to account for various sources of edge cases, in particular including different sources of anatomical or clinical variation. In the future, specific edge cases could also be integrated into model development, for instance using the synthetic minority over-sampling technique [24].

We conclude that generalizability of an algorithm is never assured, and that poor performance may be difficult to predict as individual cases may serve as unanticipated outliers. As we embrace machine and deep learning algorithms in the clinic, we must remain vigilant to potential sources of error and bias.

## 5 Disclosures

B.M. owns shares of RAYB stock.

## 6 Disclaimer

The contents do not represent the views of the U.S. Department of Veterans Affairs or the United States Government.

## Funding

R.F.T was supported by VA Career Development Award 1IK2CX002049-01.

## Declaration of Competing Interest

The authors declare that they have no known competing financial interests or personal relationships that could have appeared to influence the work reported in this paper.
