# Supplementary material for: Stress-testing pelvic autosegmentation algorithms using anatomical edge cases
Source: Phys Imaging Radiat Oncol. 2023 Jan 16;25:100413. doi: 10.1016/j.phro.2023.100413 (PMC9922913; doi:10.1016/j.phro.2023.100413)
Supplement: Supplementary data 1 [file mmc1.pdf]

Supplementary Figure 1: Overall performance of autosegmentation tools on normal and edge case cohorts. In the upper and lower panels, the distribution of 95% Hausdorff distances and mean surface distances, respectively, in millimeters (y-axis) is shown as box plots for each of three cohorts of individuals (“Normal” shown in gray, “Edge-case” with a single anatomic variant shown in pink, and “Edge-case ++” with two or more simultaneous anatomic variants shown in dark red), where each datapoint is an average across all structures for that individual. Performance is reported for each of three autosegmentation tools: atlas-based autosegmentation [AB], model-based segmentation [MB], and deep-learning based segmentation [DL]). Statistically significant differences between normal and edge case performance are denoted by asterisks, where (*) and (**) represent $p<0.05$ and $p<0.001$, respectively (Wilcoxon Rank-Sum test).

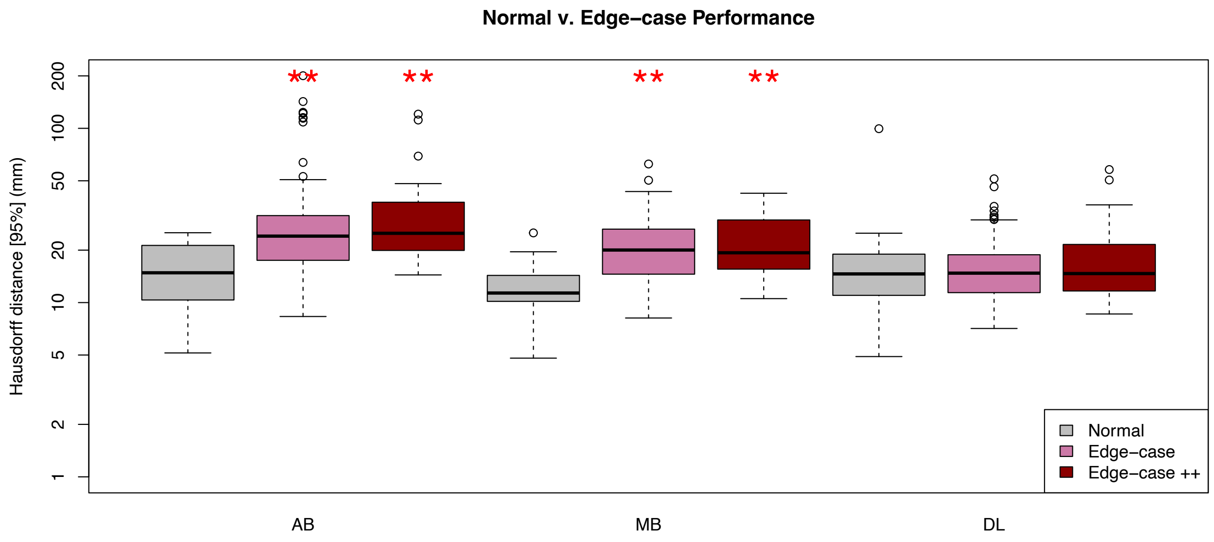
Normal v. Edge−case Performance
Hausdorff distance [95%] (mm)
200
100
50
20
10
5
2
1
**
**
**
**
Normal
Edge−case
Edge−case ++
AB
MB
DL

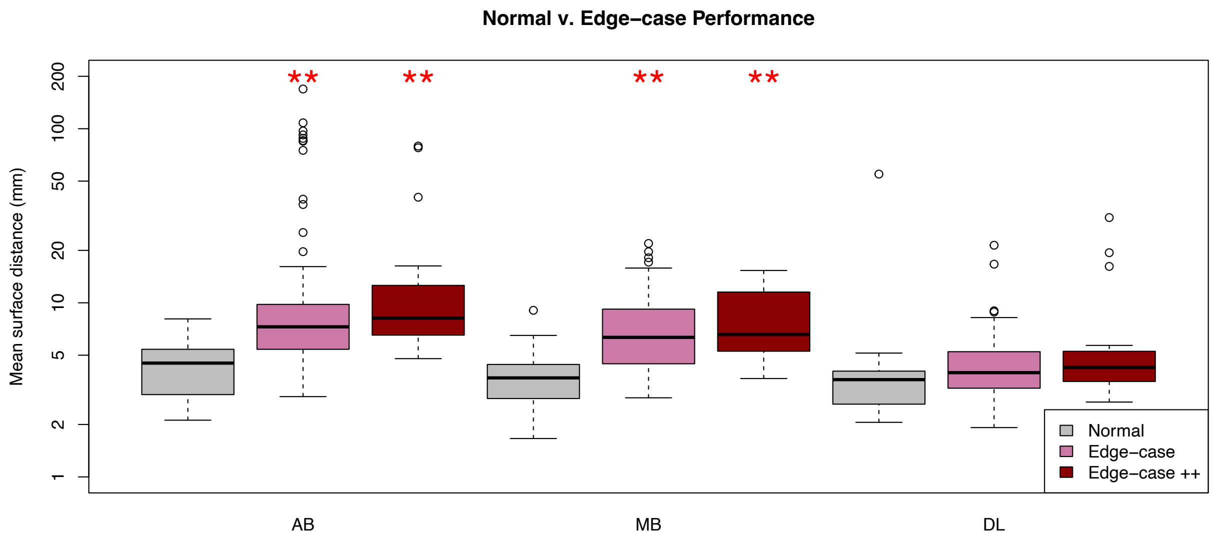
Normal v. Edge−case Performance
Mean surface distance (mm)
200
100
50
20
10
5
2
1
**
**
**
**
Normal
Edge−case
Edge−case ++
AB
MB
DL

14

Supplementary Figure 2: Structure-specific performance of deep learning autosegmentation across normal and discrete edge case cohorts. The distribution of Dice similarity coefficients (DSC, y-axis) is shown here as box plots for each of nine cohorts of individuals corresponding to "Normal" (gray), and eight distinct classes of anatomical variation defined and numbered in Methods: 1) "Hypertrophy" (blue), 2) "Droopy SVs" (white), 3) "Hardware" (light orange), 4) "Surface" (pink), 5) "High-Z Foci" (light blue), 6) "Catheter" (green), 7) "SpaceOAR" (orange), and 8) "Other" (yellow), sorted according to median DSC. Each pane corresponds to a specific structure, as labeled along y-axis, with "Femurs" representing the average result from bilateral structures. Statistically significant differences between normal and edge case performance are denoted by asterisks, where (*) and (**) represent $p<0.05$ and $p<0.001$, respectively (Wilcoxon Rank-Sum test).

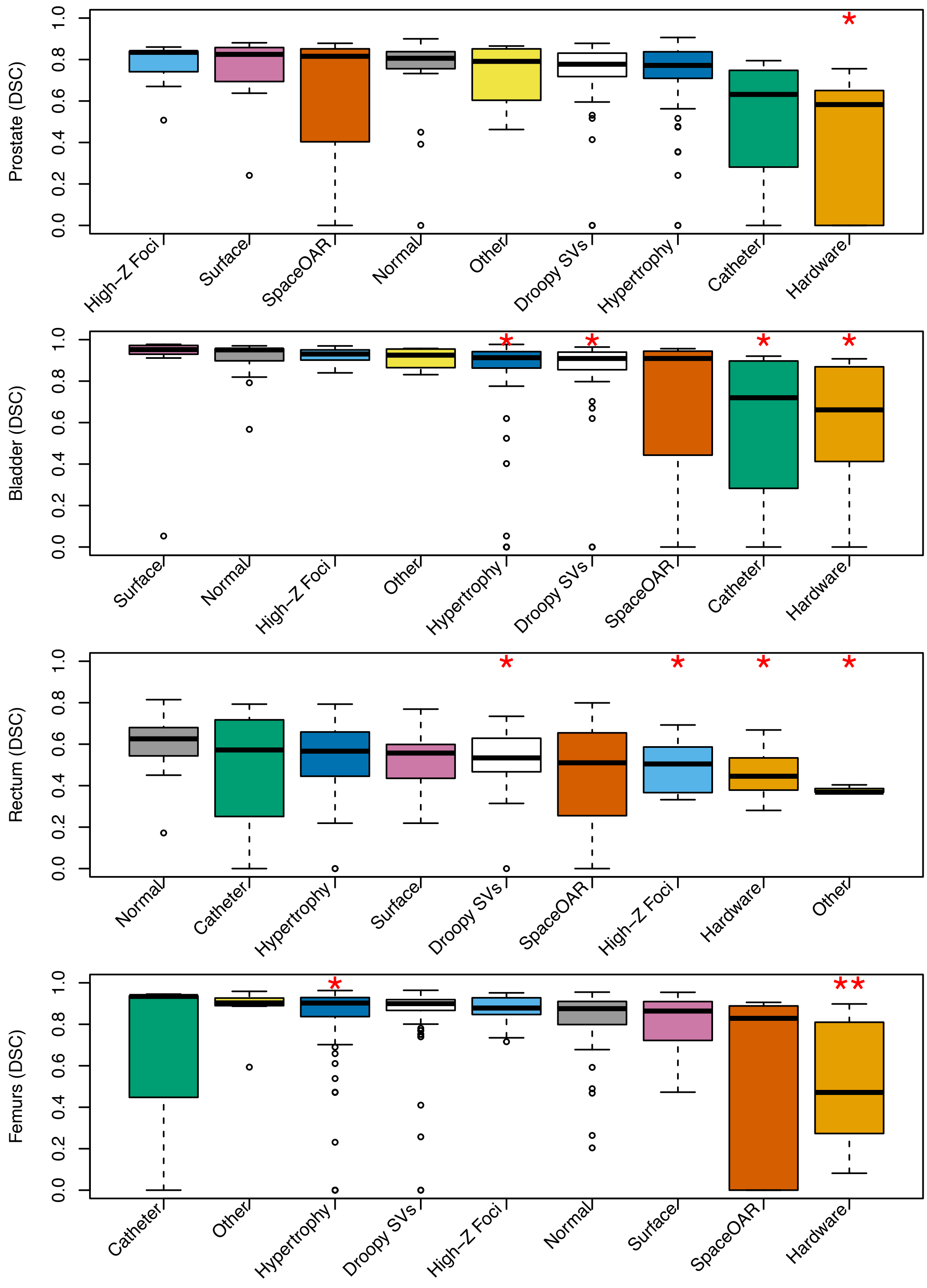
Prostate (DSC)
0.0
0.2
0.4
0.6
0.8
1.0
High−Z Foci
Surface
SpaceOAR
Normal
Other
Droopy SVs
Hypertrophy
Catheter
Hardware
Bladder (DSC)
0.0
0.2
0.4
0.6
0.8
1.0
Surface
Normal
High−Z Foci
Other
Hypertrophy
Droopy SVs
SpaceOAR
Catheter
Hardware
Rectum (DSC)
0.0
0.2
0.4
0.6
0.8
1.0
Normal
Catheter
Hypertrophy
Surface
Droopy SVs
SpaceOAR
High−Z Foci
Hardware
Other
Femurs (DSC)
0.0
0.2
0.4
0.6
0.8
1.0
Catheter
Other
Hypertrophy
Droopy SVs
High−Z Foci
Normal
Surface
SpaceOAR
Hardware

Supplementary Figure 3: Structure-specific performance of deep learning autosegmentation across normal and discrete edge case cohorts. The distribution of mean surface distances in millimeters (MSD, y-axis) is shown here as box plots for each of nine cohorts of individuals corresponding to “Normal” (gray), and eight distinct classes of anatomical variation defined and numbered in Methods: 1) “Hypertrophy” (blue), 2) “Droopy SVs” (white), 3) “Hardware” (light orange), 4) “Surface” (pink), 5) “High-Z Foci” (light blue), 6) “Catheter” (green), 7) “SpaceOAR” (orange), and 8) “Other” (yellow), sorted according to median MSD. Each pane corresponds to a specific structure, as labeled along y-axis, with “Femurs” representing the average result from bilateral structures. Statistically significant differences between normal and edge case performance are denoted by asterisks, where (*) and (**) represent $p<0.05$ and $p<0.001$, respectively (Wilcoxon Rank-Sum test).

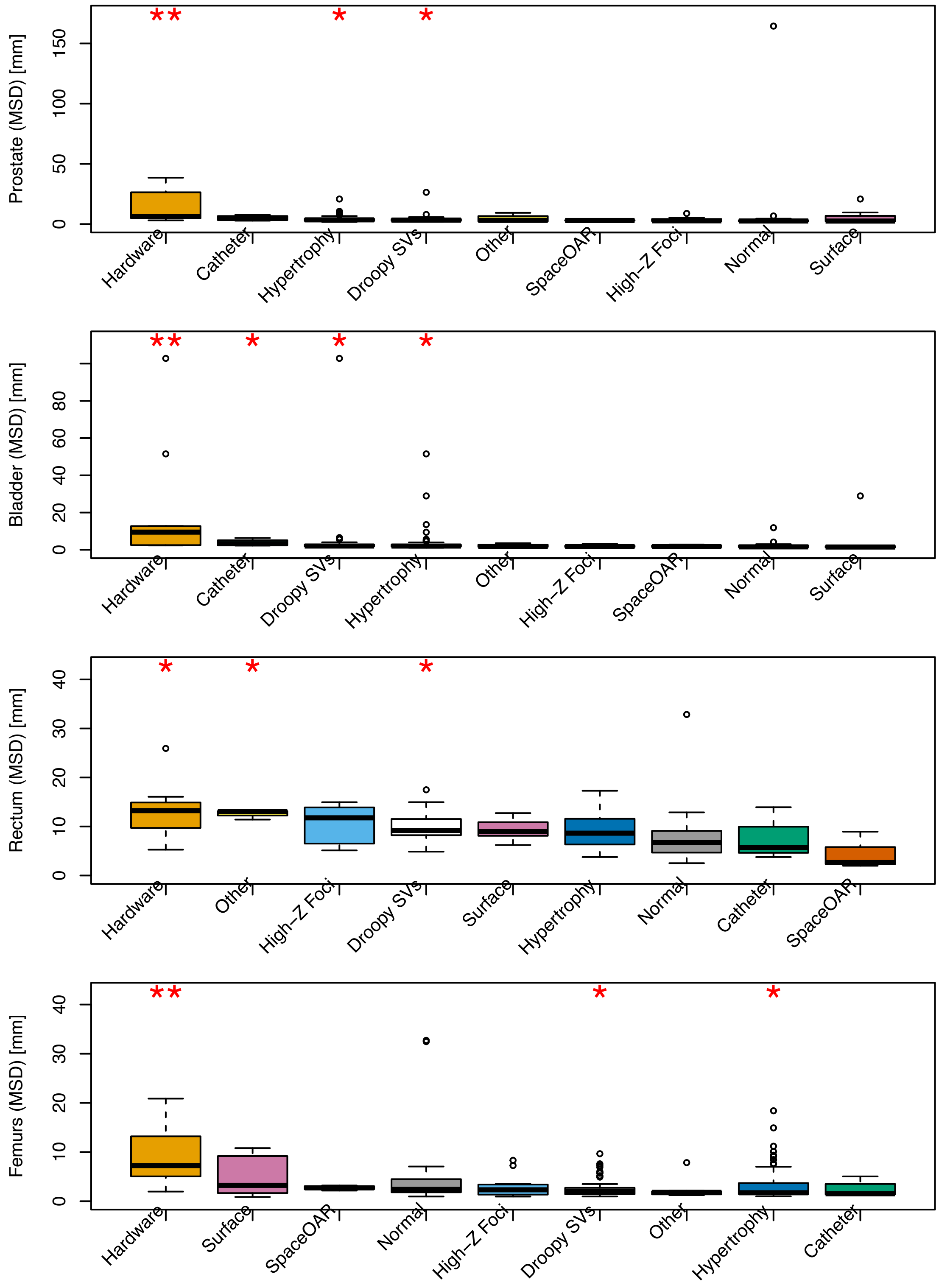
Prostate (MSD) [mm]
0
50
100
150
Hardware
Catheter
Hypertrophy
Droopy SVs
Other
SpaceOAR
High−Z Foci
Normal
Surface
Bladder (MSD) [mm]
0
20
40
60
80
Hardware
Catheter
Droopy SVs
Hypertrophy
Other
High−Z Foci
SpaceOAR
Normal
Surface
Rectum (MSD) [mm]
0
10
20
30
40
Hardware
Other
High−Z Foci
Droopy SVs
Surface
Hypertrophy
Normal
Catheter
SpaceOAR
Femurs (MSD) [mm]
0
10
20
30
40
Hardware
Surface
SpaceOAR
Normal
High−Z Foci
Droopy SVs
Other
Hypertrophy
Catheter

Supplementary Figure 4: Structure-specific performance of deep learning autosegmentation across normal and discrete edge case cohorts. The distribution of 95% Hausdorff distances in millimeters (HD 95%, y-axis) is shown here as box plots for each of nine cohorts of individuals corresponding to “Normal” (gray), and eight distinct classes of anatomical variation defined and numbered in Methods: 1) “Hypertrophy” (blue), 2) “Droopy SVs” (white), 3) “Hardware” (light orange), 4) “Surface” (pink), 5) “High-Z Foci” (light blue), 6) “Catheter” (green), 7) “SpaceOAR” (orange), and 8) “Other” (yellow), sorted according to median HD 95%. Each pane corresponds to a specific structure, as labeled along y-axis, with “Femurs” representing the average result from bilateral structures. Statistically significant differences between normal and edge case performance are denoted by asterisks, where (*) and (**) represent $p<0.05$ and $p<0.001$, respectively (Wilcoxon Rank-Sum test).

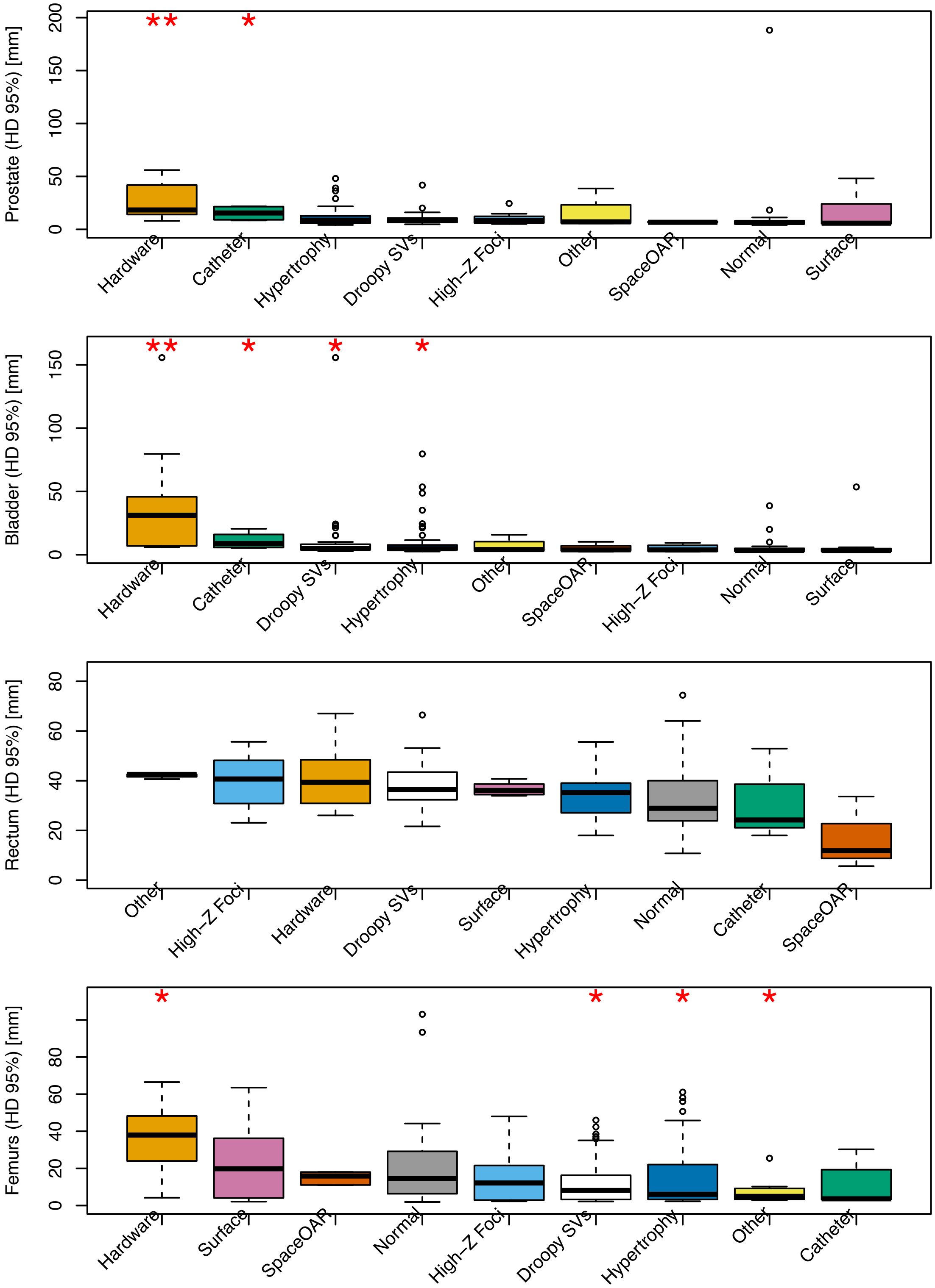
Prostate (HD 95%) [mm]
0
50
100
150
200
Hardware
Catheter
Hypertrophy
Droopy SVs
High−Z Foci
Other
SpaceOAR
Normal
Surface
Bladder (HD 95%) [mm]
0
50
100
150
Hardware
Catheter
Droopy SVs
Hypertrophy
Other
SpaceOAR
High−Z Foci
Normal
Surface
Rectum (HD 95%) [mm]
0
20
40
60
80
Other
High−Z Foci
Hardware
Droopy SVs
Surface
Hypertrophy
Normal
Catheter
SpaceOAR
Femurs (HD 95%) [mm]
0
20
40
60
80
Hardware
Surface
SpaceOAR
Normal
High−Z Foci
Droopy SVs
Hypertrophy
Other
Catheter

55

Supplementary Figure 5: Structure-specific performance of model-based autosegmentation across normal and discrete edge case cohorts. The distribution of Dice similarity coefficients (DSC, y-axis) is shown here as box plots for each of nine cohorts of individuals corresponding to "Normal" (gray), and eight distinct classes of anatomical variation defined and numbered in Methods: 1) "Hypertrophy" (blue), 2) "Droopy SVs" (white), 3) "Hardware" (light orange), 4) "Surface" (pink), 5) "High-Z Foci" (light blue), 6) "Catheter" (green), 7) "SpaceOAR" (orange), and 8) "Other" (yellow), sorted according to median DSC. Each pane corresponds to a specific structure, as labeled along y-axis, with "Femurs" representing the average result from bilateral structures. Statistically significant differences between normal and edge case performance are denoted by asterisks, where (*) and (**) represent $p<0.05$ and $p<0.001$, respectively (Wilcoxon Rank-Sum test).

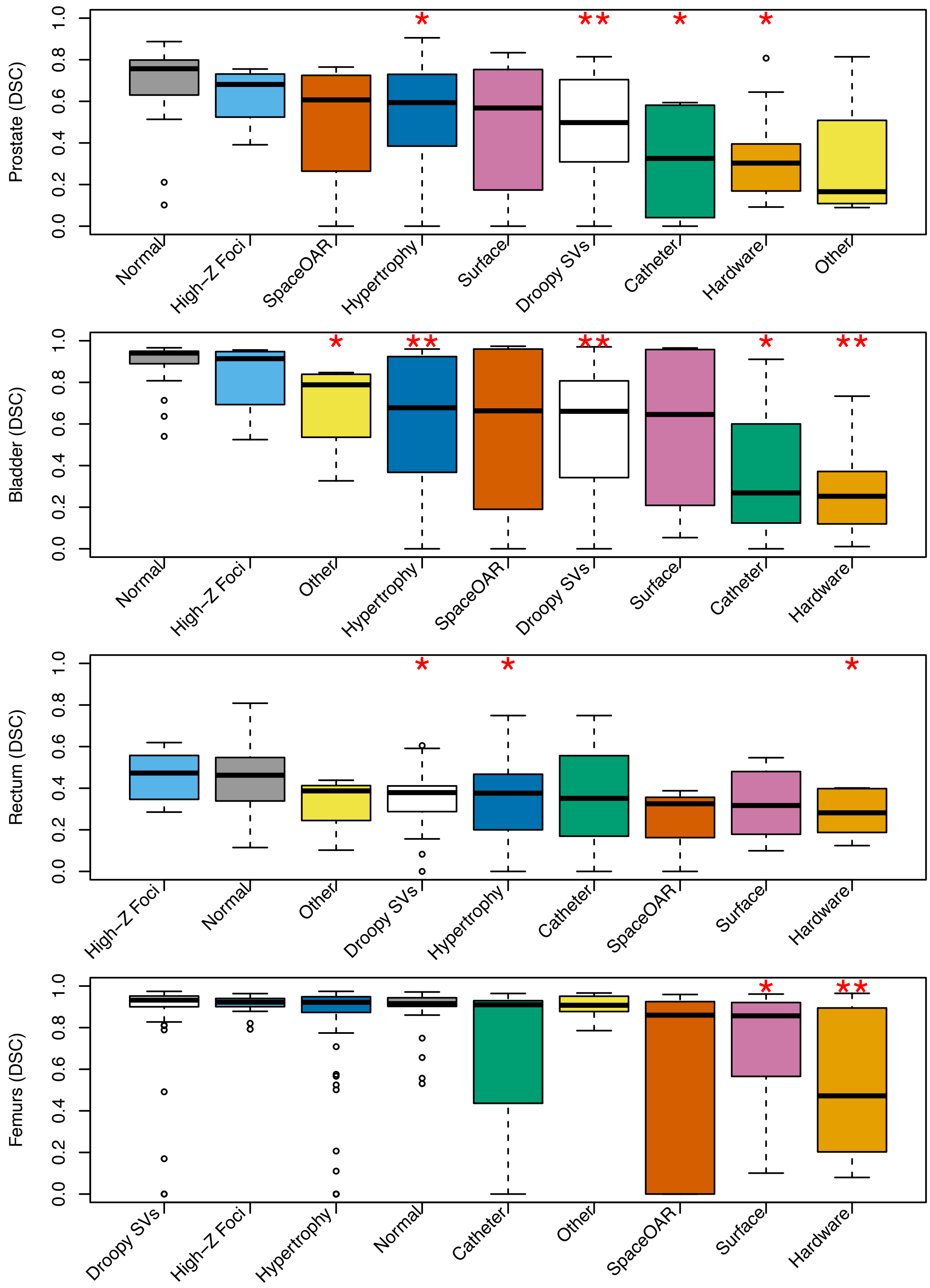
Prostate (DSC)
0.0
0.2
0.4
0.6
0.8
1.0
Normal
High–Z Foci
SpaceOAR
Hypertrophy
Surface
Droopy SVs
Catheter
Hardware
Other
Bladder (DSC)
Normal
High–Z Foci
Other
Hypertrophy
SpaceOAR
Droopy SVs
Surface
Catheter
Hardware
Rectum (DSC)
High–Z Foci
Normal
Other
Droopy SVs
Hypertrophy
Catheter
SpaceOAR
Surface
Hardware
Femurs (DSC)
Droopy SVs
High–Z Foci
Hypertrophy
Normal
Catheter
Other
SpaceOAR
Surface
Hardware

Supplementary Figure 6: Structure-specific performance of atlas-based autosegmentation across normal and discrete edge case cohorts. The distribution of Dice similarity coefficients (DSC, y-axis) is shown here as box plots for each of nine cohorts of individuals corresponding to "Normal" (gray), and eight distinct classes of anatomical variation defined and numbered in Methods: 1) "Hypertrophy" (blue), 2) "Droopy SVs" (white), 3) "Hardware" (light orange), 4) "Surface" (pink), 5) "High-Z Foci" (light blue), 6) "Catheter" (green), 7) "SpaceOAR" (orange), and 8) "Other" (yellow), sorted according to median DSC. Each pane corresponds to a specific structure, as labeled along y-axis, with "Femurs" representing the average result from bilateral structures. Statistically significant differences between normal and edge case performance are denoted by asterisks, where (*) and (**) represent $p<0.05$ and $p<0.001$, respectively (Wilcoxon Rank-Sum test).

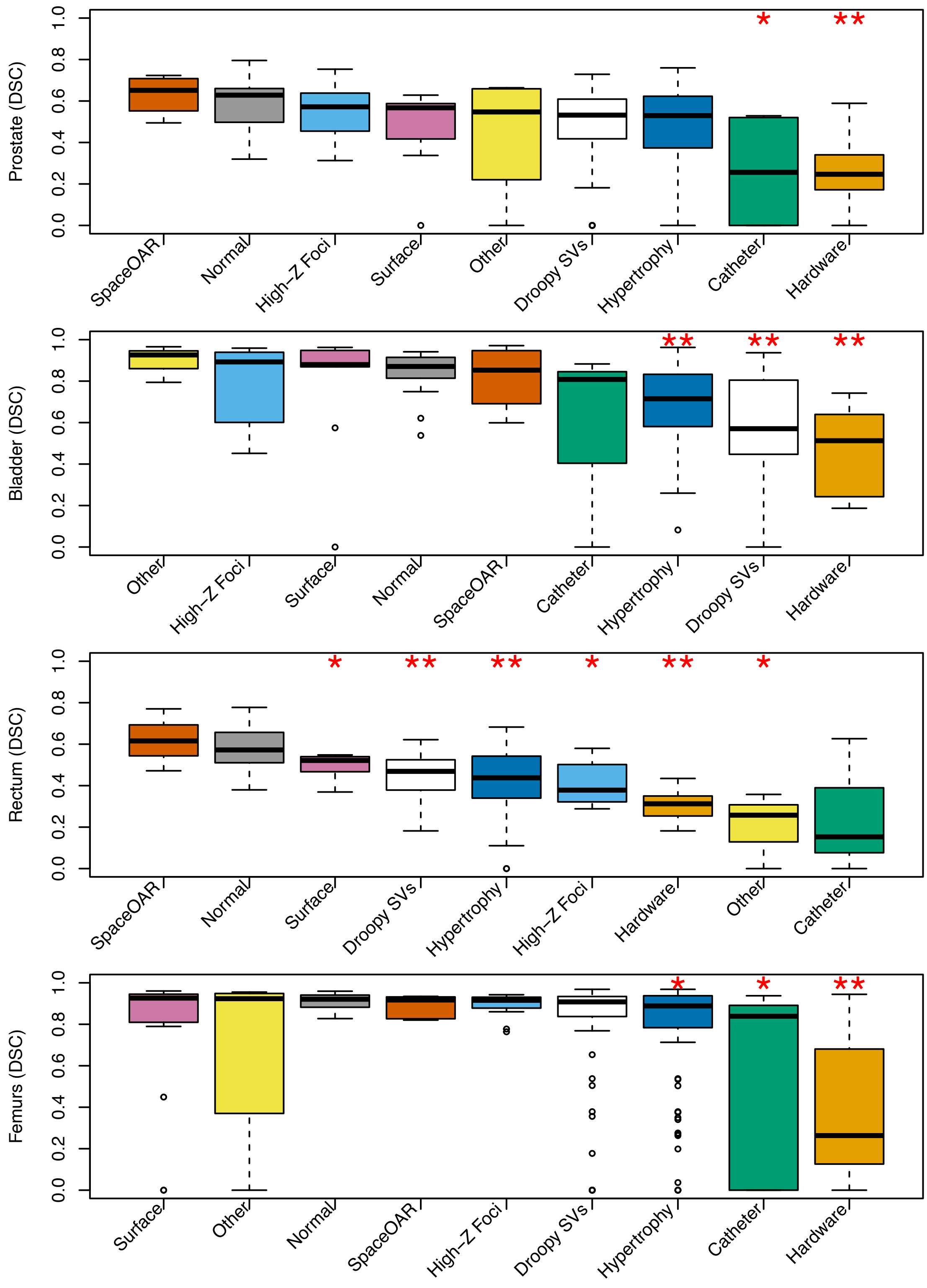
Prostate (DSC)
SpaceOAR
Normal
High–Z Foci
Surface
Other
Droopy SVs
Hypertrophy
Catheter
Hardware
Bladder (DSC)
Other
High–Z Foci
Surface
Normal
SpaceOAR
Catheter
Hypertrophy
Droopy SVs
Hardware
Rectum (DSC)
SpaceOAR
Normal
Surface
Droopy SVs
Hypertrophy
High–Z Foci
Hardware
Other
Catheter
Femurs (DSC)
Surface
Other
Normal
SpaceOAR
High–Z Foci
Droopy SVs
Hypertrophy
Catheter
Hardware

Supplementary Figure 7: Structure-specific performance of deep learning autosegmentation across normal and edge case cohorts. The distribution of Dice similarity coefficients (DSC, y-axis) is shown here as box plots for each of five structures ("Prostate", "Rectum", "Bladder", "Femur (L)", and "Femur (R)") corresponding to normal (gray) and edge cases (red). Statistically significant differences between normal and edge case performance are denoted by asterisks, where (*) represents p<0.05 (Wilcoxon Rank-Sum test).

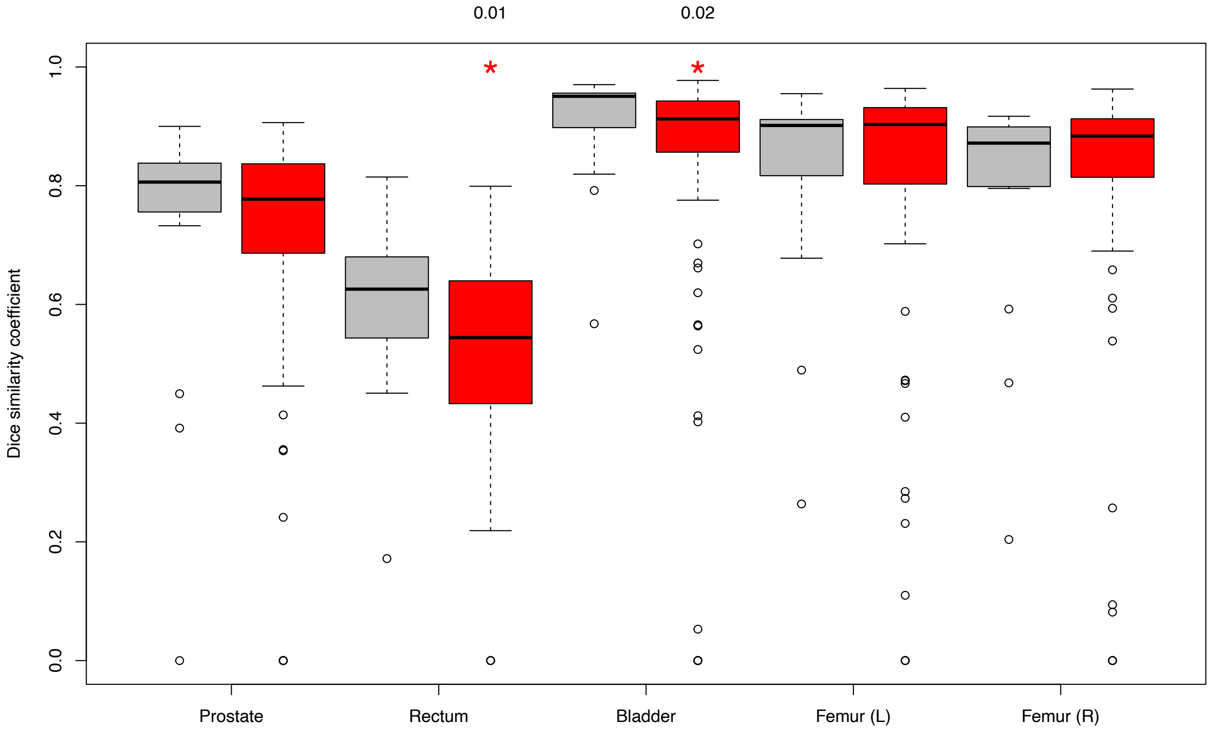

Supplementary Table 1. Characteristics of different classes of anatomical edge cases. P-values, calculated by Wilcoxon Rank-Sum test for each class of edge case v. normal, are shown where significant (<0.05). Volumes (vol.) in cubic centimeters (cm3) are median values per group.

Class	Description	Size (n)	Prostate vol. (cm3)	pval (Prostate)	Bladder vol. (cm3)	pval (Bladder)	Rectum vol. (cm3)	pval (Rectum)
	Normal	19	50.91		177.3		45.31	
1	Prostate hypertrophy	52	80.46	<0.001	125.47		32.18	0.02
2	"Droopy" seminal vesicles	37	61.8	0.03	114.86	0.04	32.89	0.04
3	Hip arthroplasty	11	53.61		72.51	<0.01	32.37	0.02
4	Prostate surface irregularity	9	63.45		225.25		32.8	
5	Calcifications	8	61.17		113.91		24.84	0.01
6	Foley catheter	4	112.66	0.04	108.45		40.04	
7	SpaceOAR	4	56.72		173.63		62.51	
8	Narrow rectum	2	55.01		245.62		26.57	
8	In-field bowel	1	50.83		61.55		24.97	
8	Morbid obesity	1	88.43		180.12		51.52	
	TOTAL	131						

Supplementary Table 2. Cohort details (individual cases). Structure volumes listed in cubic centimeters (cc).

ID	Cohort	Cohort (2)	Prostate (cc)	Bladder (cc)	Rectum (cc)
1	Prostate surface irregularity		63.45	147.53	40.01
2	"Droopy" seminal vesicles		47.60	157.43	19.05
3	"Droopy" seminal vesicles		46.98	83.62	27.26
4	Prostate hypertrophy		137.13	282.27	53.48
5	Prostate hypertrophy		72.62	119.53	72.56
6	Seeds, calcifications		83.96	158.02	16.85
7	Prostate hypertrophy		206.97	178.54	32.48
8	Normal		128.95	127.63	42.04
9	Prostate surface irregularity		50.29	360.88	15.77
10	Hip arthroplasty		38.88	87.34	33.06
11	Seeds, calcifications		69.45	104.80	55.81
12	Hip arthroplasty		57.83	72.51	44.09
13	Prostate hypertrophy		65.92	92.93	25.85
14	Prostate surface irregularity	Prostate hypertrophy	194.03	257.90	32.80
15	Prostate hypertrophy		151.35	251.53	57.33
16	Prostate hypertrophy	"Droopy" seminal vesicles	56.67	127.16	36.72
17	"Droopy" seminal vesicles		61.80	115.00	21.41
18	"Droopy" seminal vesicles		56.99	152.49	67.82
19	Prostate hypertrophy		89.11	188.66	32.29
20	Prostate surface irregularity		67.89	165.18	32.05
21	Prostate hypertrophy		84.27	110.80	18.70
22	Foley catheter		79.69	79.79	52.84
23	Prostate hypertrophy		136.43	98.19	44.30
24	In-field bowel		50.83	61.55	24.97
25	"Droopy" seminal vesicles		105.35	117.51	25.03
26	"Droopy" seminal vesicles	Hip arthroplasty	37.30	84.86	33.53
27	"Droopy" seminal vesicles		46.82	62.41	22.00
28	Hip arthroplasty		85.05	145.89	27.59
29	Foley catheter	Prostate hypertrophy	182.71	137.11	65.07
30	Seeds, calcifications		83.76	140.00	26.80
31	"Droopy" seminal vesicles		118.70	158.30	28.79
32	Prostate hypertrophy		79.70	236.66	18.52
33	Prostate hypertrophy		43.90	175.42	19.53
34	Prostate hypertrophy		166.90	114.33	17.79
35	Hip arthroplasty		54.18	63.12	27.03
36	Prostate hypertrophy		197.61	49.63	30.54
37	Prostate hypertrophy		75.19	332.99	29.63
38	Prostate hypertrophy	"Droopy" seminal vesicles	50.53	204.27	60.23
39	Seeds, calcifications		39.44	70.50	26.16
40	"Droopy" seminal vesicles		85.56	60.61	17.36
41	Foley catheter		43.81	73.99	27.25
42	Seeds, calcifications		51.46	61.56	23.52
43	"Droopy" seminal vesicles		48.03	66.74	16.66
44	Narrow rectum		63.61	254.20	32.47
45	Prostate hypertrophy	"Droopy" seminal vesicles	80.98	176.19	26.01
46	Prostate hypertrophy		94.34	122.04	17.10
47	"Droopy" seminal vesicles		65.38	242.32	50.79
48	"Droopy" seminal vesicles		85.76	295.42	24.34
49	Hip arthroplasty		36.97	103.59	23.62
50	"Droopy" seminal vesicles		95.36	114.86	43.57
51	Prostate surface irregularity	Prostate hypertrophy	151.46	507.35	28.31
52	Prostate hypertrophy		79.80	483.27	43.63
53	Seeds, calcifications		34.73	122.26	18.42
54	"Droopy" seminal vesicles		40.56	52.72	40.11
55	Prostate surface irregularity		75.66	348.29	90.39
56	Prostate hypertrophy		106.95	234.02	32.96
57	Prostate hypertrophy		47.87	90.25	49.44
58	Prostate surface irregularity		63.31	115.29	27.73
59	"Droopy" seminal vesicles		45.17	222.83	23.23
60	Prostate hypertrophy		96.76	162.70	18.09
61	Prostate hypertrophy		72.00	133.58	27.03
62	Prostate hypertrophy		53.57	129.84	18.80
63	"Droopy" seminal vesicles		96.04	71.08	56.81
64	Normal		57.90	102.95	23.29
65	Prostate hypertrophy		111.19	433.62	28.72
66	Prostate hypertrophy		53.43	54.13	29.63
67	Prostate hypertrophy	"Droopy" seminal vesicles	80.77	101.61	36.33
68	Prostate hypertrophy	"Droopy" seminal vesicles	58.58	190.80	37.47
69	Prostate hypertrophy		92.16	174.26	39.31
70	"Droopy" seminal vesicles	Hip arthroplasty	47.16	61.65	36.02
71	Hip arthroplasty		90.71	44.90	22.70
72	"Droopy" seminal vesicles		52.89	78.49	44.91
73	Normal		119.76	91.47	60.34
74	Prostate hypertrophy	Hip arthroplasty	53.61	71.37	30.28
75	"Droopy" seminal vesicles		57.35	234.30	59.89
76	Prostate hypertrophy		61.98	117.05	20.50
77	Prostate hypertrophy	"Droopy" seminal vesicles	55.16	100.78	57.48
78	Prostate surface irregularity		37.60	113.00	344.90
79	Prostate hypertrophy	"Droopy" seminal vesicles	86.07	78.74	42.40
80	Prostate hypertrophy	"Droopy" seminal vesicles	46.06	68.74	23.37
81	"Droopy" seminal vesicles		66.34	78.58	27.51
82	Narrow rectum		46.42	237.03	20.67
83	"Droopy" seminal vesicles		75.60	251.43	32.94
84	Seeds, calcifications		61.97	105.69	29.04
85	Prostate hypertrophy		56.40	86.47	30.02
86	Prostate surface irregularity		49.62	225.22	48.15
87	"Droopy" seminal vesicles		56.68	156.66	21.77
88	Prostate hypertrophy		120.75	69.87	40.62
89	"Droopy" seminal vesicles		80.14	160.11	25.38
90	Prostate hypertrophy		80.14	74.78	34.53
91	Normal		35.49	177.41	28.45
92	"Droopy" seminal vesicles		79.89	83.01	32.89
93	Prostate hypertrophy		115.34	47.50	63.81
94	"Droopy" seminal vesicles		125.60	85.65	30.66
95	"Droopy" seminal vesicles	Hip arthroplasty	99.86	205.13	32.37
96	"Droopy" seminal vesicles		55.14	51.55	28.99
97	Seeds, calcifications		60.37	122.13	22.93
98	Prostate hypertrophy		125.48	192.96	32.06
99	Prostate hypertrophy		63.87	123.79	23.54
100	Prostate hypertrophy		42.35	86.08	23.18
101	"Droopy" seminal vesicles		63.97	249.39	283.74
102	Normal		54.55	44.09	28.60
103	Hip arthroplasty		43.66	53.83	37.27
104	Prostate hypertrophy	"Droopy" seminal vesicles	73.62	54.44	44.95
105	Prostate hypertrophy		51.00	74.35	27.98
106	Prostate hypertrophy		42.55	56.43	48.48
107	Prostate hypertrophy		65.90	171.13	23.05
108	Prostate hypertrophy		81.56	93.68	20.76
109	Normal		31.92	130.16	42.33
110	Normal		62.22	246.52	86.16
111	Normal		66.95	133.91	87.26
112	Normal		64.31	99.51	22.67
113	Normal		50.20	258.12	41.15
114	Normal		61.68	272.42	54.52
115	SpaceOAR		60.85	689.82	67.81
116	Normal		66.84	209.13	53.23
117	Normal		35.36	76.36	56.43
118	Normal		30.29	178.50	21.07
119	Prostate hypertrophy		98.91	630.80	40.11
120	Normal		40.18	177.30	67.20
121	SpaceOAR		109.66	143.22	57.21
122	SpaceOAR		43.47	204.03	29.81
123	Prostate hypertrophy		53.08	98.81	136.07
124	Normal		27.20	281.91	34.15
125	Normal		41.13	375.57	48.68
126	Normal		50.91	216.65	45.31
127	Prostate hypertrophy		153.81	174.37	73.21
128	Morbid obesity		88.43	180.12	51.52
129	Normal		42.12	113.28	67.47
130	SpaceOAR		52.58	130.23	109.78
131	Foley catheter	Prostate hypertrophy	145.63	140.90	24.60

Supplementary Table 3. Detailed list of structure comparisons per individual for prostate, rectum, bladder, and bilateral femoral heads (femur_L, femur_R). Comparisons all versus expert clinician-delineated contours via Dice similarity coefficient (DSC). Results are for model-based (MB), deep learning (DL), and atlas-based (AB) algorithms.

ID	Prostate			Rectum			Bladder			Femur_L			Femur_R		
	MB	DL	AB	MB	DL	AB	MB	DL	AB	MB	DL	AB	MB	DL	AB
1	0.07	0.86	0.57	0.10	0.55	0.52	0.11	0.95	0.95	0.10	0.92	0.96	0.32	0.90	0.95
2	0.03	0.82	0.71	0.16	0.47	NA	0.22	0.93	0.64	0.89	0.95	0.93	0.94	0.93	0.95
3	0.63	0.81	0.52	0.40	0.47	0.41	0.48	0.88	0.58	0.91	0.96	0.95	0.95	0.92	0.96
4	0.86	0.91	0.41	0.47	0.66	0.68	0.94	0.96	0.85	0.93	0.92	0.94	0.96	0.90	0.95
5	0.64	0.72	0.00	0.48	0.63	0.42	0.85	0.93	NA	0.91	0.85	0.27	0.90	0.84	0.04
6	0.47	0.51	0.47	0.31	0.33	0.31	0.52	0.91	0.47	0.89	0.95	0.94	0.93	0.92	0.92
7	0.46	0.82	NA	0.65	0.68	0.52	0.27	0.91	NA	0.95	0.90	0.78	0.96	0.87	0.28
8	0.65	0.73	0.38	0.28	0.66	0.51	0.91	0.86	0.84	0.93	0.91	0.93	0.94	0.91	0.93
9	0.75	0.84	0.47	0.17	0.38	0.37	0.96	0.98	0.87	0.82	0.72	0.79	0.83	0.73	0.82
10	NA	NA	0.42	NA	NA	0.31	NA	NA	0.24	NA	NA	0.94	NA	NA	NA
11	0.76	0.84	0.44	0.56	0.62	0.52	0.93	0.92	0.73	0.91	0.91	0.94	0.94	0.89	0.93
12	0.21	0.61	0.25	0.40	0.52	0.43	0.12	0.66	NA	0.87	0.78	0.80	NA	NA	NA
13	0.68	0.82	0.00	0.44	0.55	0.11	0.32	0.86	NA	0.94	0.91	0.00	0.91	0.86	0.00
14	0.28	0.24	0.34	0.19	0.22	0.54	0.05	0.05	0.90	0.50	0.47	0.92	0.57	0.54	0.94
15	0.68	0.84	0.76	0.50	0.70	0.52	0.95	0.95	0.71	0.90	0.92	0.53	0.96	0.89	0.81
16	0.75	0.83	0.00	0.41	0.56	0.31	0.82	0.92	NA	0.96	0.93	0.54	0.91	0.88	0.38
17	0.70	0.74	0.55	0.17	0.31	0.33	0.92	0.92	0.56	0.93	0.96	0.94	0.97	0.89	0.95
18	0.71	0.76	0.18	0.41	0.73	NA	0.94	0.94	0.62	0.93	0.91	0.91	0.89	0.87	0.90
19	0.79	0.80	0.46	0.36	0.62	0.45	0.90	0.94	0.84	0.95	0.89	0.90	0.96	0.89	0.95
20	0.69	0.88	0.58	0.45	0.57	0.48	0.56	0.95	0.87	0.56	0.70	0.45	0.91	0.85	0.91
21	0.76	0.82	0.58	0.13	0.56	0.47	0.95	0.91	0.90	0.86	0.95	0.92	0.90	0.95	0.92
22	0.57	0.70	0.00	0.36	0.64	0.00	0.25	0.57	NA	0.91	0.93	0.00	0.91	0.94	NA
23	0.82	0.73	NA	0.25	0.64	0.00	0.46	0.78	NA	0.92	0.95	0.38	0.95	0.93	0.20
24	0.81	0.84	0.00	0.39	0.40	0.00	0.83	0.90	NA	0.90	0.94	0.00	0.92	0.96	0.00
25	0.52	0.83	NA	0.44	0.51	NA	0.74	0.92	NA	0.84	0.74	0.82	0.79	0.75	0.50
26	0.32	NA	0.00	0.29	NA	0.18	0.34	NA	NA	0.94	NA	0.00	NA	NA	NA
27	0.33	0.68	0.44	0.33	0.59	0.41	0.00	0.86	0.47	0.94	0.89	0.93	0.94	0.87	0.93
28	0.09	0.00	0.25	0.28	0.54	0.32	0.70	0.56	0.63	0.34	0.47	0.30	0.20	0.09	0.14
29	0.59	0.56	0.51	0.75	0.79	0.63	0.91	0.92	0.81	0.87	0.95	0.92	0.94	0.94	0.94
30	0.58	0.67	0.31	0.56	0.55	0.35	0.90	0.96	0.91	0.88	0.94	0.93	0.91	0.95	0.94
31	0.50	0.76	0.46	0.39	0.53	0.50	0.96	0.95	0.92	0.95	0.87	0.92	0.94	0.86	0.93
32	0.38	0.88	0.73	0.37	0.43	0.40	0.06	0.97	0.79	0.84	0.93	0.90	0.86	0.96	0.86
33	0.63	0.85	0.00	0.32	0.52	NA	0.58	0.94	NA	0.92	0.90	0.00	0.95	0.86	NA
34	0.46	0.76	0.63	0.25	0.32	0.35	0.96	0.94	0.71	0.77	0.70	0.73	0.78	0.69	0.77
35	0.17	0.67	0.23	0.40	0.32	0.30	0.01	0.87	NA	0.09	0.27	0.23	0.96	0.89	0.93
36	0.65	0.35	0.24	0.45	0.62	0.57	0.59	0.40	0.46	0.93	0.83	0.88	0.89	0.81	0.87
37	0.02	0.73	0.00	0.20	0.56	0.43	0.96	0.95	0.08	0.88	0.93	0.88	0.91	0.94	0.71
38	0.53	0.85	0.65	0.51	0.67	0.33	0.43	0.88	0.43	0.90	0.90	0.77	0.95	0.90	0.80
39	0.73	0.83	0.54	0.40	0.53	0.41	0.94	0.89	0.93	0.96	0.94	0.92	0.94	0.87	0.93
40	0.50	0.53	0.00	0.08	0.35	NA	0.91	0.70	0.00	0.84	0.75	0.00	0.81	0.77	0.00
41	0.08	0.79	0.00	0.34	0.50	NA	0.29	0.87	0.00	0.92	0.93	0.00	0.96	0.90	0.00
42	0.39	0.81	0.75	0.62	0.38	0.29	0.54	0.84	0.45	0.96	0.88	0.89	0.91	0.86	0.88
43	0.00	0.00	0.53	0.00	0.00	NA	0.00	0.00	0.48	0.00	0.00	0.90	0.00	0.00	0.89
44	0.09	0.87	0.65	0.10	0.37	0.26	0.75	0.95	0.93	0.87	0.91	0.94	0.95	0.89	0.95
45	0.17	0.85	0.53	0.39	0.44	0.42	0.67	0.95	0.81	0.93	0.89	0.95	0.95	0.88	0.96
46	0.41	0.72	0.51	0.10	0.23	0.29	0.84	0.88	0.75	0.80	0.73	0.80	0.71	0.66	0.75
47	0.47	0.84	0.59	0.59	0.68	0.62	0.94	0.94	0.94	0.96	0.88	0.90	0.95	0.87	0.93
48	0.51	0.79	0.45	0.16	0.46	0.38	0.53	0.95	0.69	0.91	0.90	0.94	0.94	0.90	0.94
49	0.11	0.00	0.21	0.12	0.28	0.25	0.37	0.41	0.64	0.08	0.11	0.10	0.17	0.08	0.11
50	0.31	0.73	0.53	0.37	0.62	0.43	0.79	0.93	0.81	0.88	0.95	0.85	0.96	0.96	0.85
51	0.75	0.64	0.42	0.47	0.49	0.43	0.96	0.98	0.96	0.93	0.92	0.93	0.96	0.88	0.94
52	0.10	0.85	0.68	0.20	0.55	NA	0.80	0.96	0.76	0.88	0.77	0.81	0.78	0.70	0.77
53	0.73	0.84	0.65	0.29	0.36	0.33	0.96	0.97	0.95	0.82	0.73	0.76	0.79	0.72	0.78
54	0.00	0.78	0.67	0.22	0.64	0.53	0.06	0.85	0.43	0.95	0.87	0.90	0.94	0.88	0.86
55	0.00	0.75	0.62	0.49	0.77	0.55	0.73	0.97	0.88	0.89	0.95	0.94	0.94	0.92	0.95
56	0.72	0.72	0.52	0.53	0.62	0.54	0.94	0.95	0.93	0.88	0.91	0.95	0.94	0.88	0.95
57	NA	NA	NA	0.43	0.69	0.61	0.93	0.92	0.75	0.96	0.94	0.91	0.93	0.89	0.82
58	NA	NA	0.63	NA	NA	0.53	NA	NA	0.57	NA	NA	0.96	NA	NA	0.95
59	0.46	0.83	0.57	0.41	0.56	0.48	0.97	0.96	0.92	0.91	0.80	0.83	0.86	0.78	0.83
60	0.00	0.00	0.60	0.00	0.00	0.34	0.00	0.00	0.81	0.00	0.00	0.94	0.00	0.00	0.94
61	0.73	0.80	NA	0.37	0.53	0.46	0.96	0.92	NA	0.92	0.92	0.85	0.94	0.93	0.86
62	0.83	0.89	0.68	0.35	0.43	0.39	0.67	0.91	0.60	0.92	0.92	0.95	0.95	0.90	0.93
63	0.68	0.79	0.62	0.42	0.52	0.49	0.03	0.82	0.49	0.96	0.90	0.91	0.93	0.86	0.90
64	0.79	0.80	0.50	0.46	0.57	0.56	0.91	0.94	0.87	0.91	0.91	0.93	0.97	0.88	0.94
65	0.20	0.76	0.09	0.20	0.53	0.38	0.56	0.97	0.57	0.87	0.96	0.89	0.90	0.93	0.87
66	0.55	0.83	0.56	0.40	0.51	0.54	0.31	0.93	0.61	0.91	0.93	0.95	0.91	0.93	0.91
67	0.39	0.86	0.70	0.41	0.49	0.47	0.64	0.91	0.71	0.96	0.92	0.92	0.94	0.91	0.94
68	0.77	0.76	0.55	0.45	0.61	0.55	0.64	0.94	0.26	0.93	0.93	0.89	0.90	0.84	0.88
69	0.37	0.47	0.16	0.48	0.29	0.17	0.66	0.84	0.70	0.78	0.77	0.76	0.80	0.77	0.80
70	0.28	0.00	0.59	0.25	0.53	0.40	0.29	0.00	0.19	0.17	0.41	0.18	0.49	0.26	0.36
71	0.81	0.58	0.27	0.38	0.40	0.35	0.14	0.85	0.74	0.45	0.59	0.56	0.94	0.88	0.93
72	0.80	0.88	0.73	0.31	0.70	0.50	0.59	0.91	0.53	0.88	0.92	0.92	0.91	0.86	0.91
73	0.10	0.45	0.32	0.42	0.63	NA	0.64	0.79	0.54	0.94	0.85	0.89	0.93	0.87	0.92
74	0.64	0.36	0.41	0.18	0.45	0.19	0.22	0.00	0.39	0.21	0.47	0.26	0.52	0.61	0.50
75	0.76	0.77	0.57	0.39	0.58	0.56	0.77	0.88	0.80	0.93	0.95	0.91	0.96	0.90	0.92
76	0.53	0.78	0.62	0.45	0.59	0.41	0.91	0.90	0.55	0.96	0.93	0.88	0.95	0.94	0.92
77	0.46	0.79	0.60	0.60	0.65	0.48	0.68	0.89	0.62	0.96	0.90	0.92	0.96	0.91	0.92
78	0.45	0.81	0.00	0.19	0.61	0.47	0.31	0.91	0.00	0.91	0.89	0.00	0.93	0.88	0.00
79	0.20	0.85	0.46	0.29	0.66	0.33	0.22	0.91	0.33	0.90	0.95	0.94	0.97	0.93	0.95
80	0.63	0.77	0.53	0.16	0.35	0.36	0.29	0.86	0.67	0.95	0.90	0.94	0.97	0.91	0.97
81	0.75	0.73	0.43	0.41	0.43	0.52	0.77	0.84	0.83	0.90	0.92	0.93	0.94	0.87	0.92
82	0.13	0.74	0.66	0.44	0.37	0.36	0.33	0.96	0.97	0.95	0.90	0.95	0.97	0.89	0.96
83	0.80	0.87	0.63	0.47	0.51	0.54	0.96	0.94	0.93	0.90	0.95	0.95	0.95	0.91	0.95
84	0.64	0.84	0.60	0.54	0.69	0.58	0.84	0.94	0.87	0.94	0.84	0.87	0.93	0.85	0.91
85	0.68	0.76	0.00	0.46	0.57	0.00	0.96	0.90	NA	0.90	0.80	0.00	0.84	0.78	0.00
86	0.83	0.86	0.59	0.55	0.59	0.54	0.97	0.95	0.95	0.87	0.76	0.81	0.85	0.76	0.83
87	0.19	0.85	0.00	0.37	0.48	0.59	0.73	0.94	NA	0.90	0.91	0.85	0.93	0.90	0.65
88	0.77	0.74	0.31	0.34	0.66	0.63	0.25	0.89	0.52	0.81	0.74	0.82	0.82	0.75	0.85
89	0.64	0.72	0.41	0.34	0.44	0.43	0.66	0.95	0.52	0.85	0.77	0.85	0.81	0.74	0.84
90	0.48	0.76	0.54	0.68	0.65	0.64	0.92	0.87	0.80	0.93	0.93	0.88	0.95	0.86	0.86
91	0.78	0.78	0.66	0.37	0.49	0.49	0.94	0.96	0.85	0.91	0.81	0.88	0.91	0.80	0.89
92	0.81	0.60	0.00	0.44	0.57	NA	0.64	0.67	0.00	0.93	0.94	0.00	0.95	0.93	0.00
93	0.91	0.48	0.32	0.50	0.78	NA	0.71	0.52	0.46	0.89	0.94	0.91	0.94	0.95	0.91
94	0.22	0.41	0.40	0.32	0.65	0.60	0.38	0.80	0.47	0.93	0.91	0.93	0.95	0.90	0.93
95	0.33	0.65	0.00	0.40	0.38	NA	0.73	0.91	NA	0.83	0.90	0.00	0.89	0.81	0.00
96	0.71	0.71	0.61	0.31	0.59	0.36	0.81	0.90	0.47	0.92	0.93	0.94	0.95	0.91	0.95
97	0.72	0.86	0.62	0.38	0.48	0.48	0.95	0.94	0.96	0.93	0.88	0.86	0.91	0.84	0.88
98	0.73	0.88	NA	0.38	0.67	0.59	0.77	0.93	NA	0.93	0.93	0.38	0.95	0.92	0.34
99	0.65	0.70	0.57	0.28	0.38	0.31	0.29	0.86	0.64	0.96	0.88	0.90	0.96	0.89	0.95
100	0.78	0.81	0.75	0.46	0.54	0.57	0.96	0.93	0.89	0.89	0.94	0.96	0.95	0.92	0.96
101	0.31	0.82	0.55	NA	NA	NA	0.95	0.96	0.83	0.96	0.90	0.91	0.95	0.88	0.93
102	0.81	0.73	0.57	0.53	0.62	0.48	0.54	0.82	0.75	0.90	0.83	0.87	0.87	0.80	0.86
103	0.40	0.76	0.14	0.19	0.67	0.34	0.06	0.87	NA	0.26	0.28	0.19	0.93	0.89	0.87
104	0.35	0.52	0.68	0.31	0.66	0.52	0.00	0.62	0.31	0.97	0.90	0.90	NA	NA	NA
105	0.46	0.75	0.66	0.20	0.66	0.38	0.76	0.85	0.62	0.92	0.91	0.97	0.94	0.91	0.93
106	0.60	0.83	0.73	0.53	0.65	0.63	0.41	0.93	0.85	0.94	0.94	0.96	0.95	0.92	0.94
107	0.32	0.82	0.51	0.25	0.61	0.48	0.93	0.94	0.93	0.93	0.93	0.95	0.96	0.91	0.95
108	0.52	0.58	0.43	0.15	0.44	0.44	0.65	0.89	0.67	0.11	0.23	0.35	0.79	0.76	0.78
109	0.84	0.84	0.63	0.81	0.75	0.63	0.95	0.95	0.94	0.95	0.91	0.91	0.96	0.90	0.95
110	0.83	0.00	0.74	0.55	0.17	0.74	0.97	0.57	0.94	0.97	0.26	0.91	0.93	0.20	0.92
111	0.79	0.81	0.54	NA	NA	NA	0.95	0.93	0.91	0.92	0.93	0.96	0.93	0.92	0.95
112	0.89	0.90	0.65	0.55	0.54	0.57	0.95	0.96	0.88	0.87	0.95	0.92	0.95	0.92	0.96
113	0.59	0.78	0.55	0.43	0.67	0.55	0.94	0.95	0.88	0.75	0.68	0.94	0.66	0.59	0.93
114	0.79	0.86	0.79	0.70	0.79	0.78	0.81	0.96	0.92	0.91	0.91	0.92	0.97	0.89	0.95
115	0.53	0.82	0.49	NA	NA	NA	0.38	0.89	0.60	NA	NA	NA	NA	NA	NA
116	0.71	0.87	0.63	0.62	0.65	0.66	0.96	0.96	0.84	0.91	0.90	0.94	0.95	0.87	0.94
117	0.76	0.80	0.79	0.67	0.79	0.71	0.87	0.85	0.86	0.91	0.82	0.85	0.89	0.82	0.88
118	0.51	0.83	0.64	0.33	0.45	NA	0.96	0.95	0.90	0.90	0.96	0.95	0.95	0.90	0.95
119	0.74	0.90	0.54	0.15	0.68	NA	0.46	0.97	0.90	0.92	0.94	0.94	0.93	0.93	0.89
120	0.66	0.81	0.80	0.48	0.60	0.58	0.95	0.95	0.94	0.91	0.79	0.83	0.86	0.80	0.86
121	0.76	0.88	0.61	0.39	0.80	0.62	0.95	0.93	0.92	0.93	0.91	0.93	0.96	0.89	0.92
122	0.69	0.81	0.72	0.33	0.51	0.47	0.97	0.96	0.97	0.88	0.83	0.82	0.84	0.82	0.83
123	0.50	0.85	0.63	NA	NA	NA	0.94	0.95	0.81	0.95	0.89	0.94	0.58	0.89	0.95
124	0.61	0.82	0.36	0.11	0.68	0.38	0.94	0.96	0.62	0.90	0.91	0.93	0.93	0.90	0.89
125	0.74	0.86	0.49	0.24	0.81	0.63	0.93	0.97	0.79	0.90	0.91	0.96	0.93	0.90	0.94
126	0.21	0.39	0.49	0.34	0.53	NA	0.94	0.96	0.78	0.56	0.49	0.85	0.53	0.47	0.85
127	0.12	0.69	0.59	NA	NA	NA	0.69	0.86	0.82	0.91	0.81	0.82	0.84	0.78	0.82
128	0.20	0.46	0.44	NA	NA	NA	0.85	0.83	0.79	0.88	0.90	0.91	0.79	0.59	0.74
129	0.86	0.83	0.66	NA	NA	NA	0.71	0.95	0.94	0.95	0.85	0.88	0.92	0.85	0.90
130	0.00	0.00	0.69	0.00	0.00	0.77	0.00	0.00	0.78	0.00	0.00	0.91	0.00	0.00	0.93
131	0.00	0.00	0.53	0.00	0.00	0.15	0.00	0.00	0.88	0.00	0.00	0.84	0.00	0.00	0.86

Supplementary Table 4. Detailed list of structure comparisons per individual for prostate, rectum, bladder, and bilateral femoral heads (femur_L, femur_R). Comparisons all versus clinician-delineated contours via Mean surface distance (MSD) in millimeters. Results are for model-based (MB), deep learning (DL), and atlas-based (AB) algorithms.

ID	Prostate			Rectum			Bladder			Femur_L			Femur_R		
	MB	DL	AB	MB	DL	AB	MB	DL	AB	MB	DL	AB	MB	DL	AB
1	21.44	2.34	6.01	18.94	8.04	11.30	22.83	1.40	1.46	19.63	1.42	0.83	15.40	1.67	1.00
2	18.83	3.01	4.03	13.94	8.47	NA	14.70	2.02	16.04	2.32	1.07	1.34	1.37	1.32	1.17
3	5.50	2.52	5.09	7.31	10.81	15.65	6.99	2.51	13.87	1.82	0.96	0.93	1.28	1.48	0.85
4	3.34	2.20	9.40	9.37	7.14	7.90	1.89	1.57	5.06	1.51	1.72	1.59	1.27	2.02	1.28
5	5.63	4.15	62.33	11.53	6.57	9.57	2.63	1.82	NA	1.55	3.06	9.73	1.61	3.83	19.72
6	11.71	8.84	9.31	9.66	14.95	21.76	11.77	2.27	9.63	2.50	0.95	0.90	1.46	1.38	1.22
7	14.14	4.13	NA	4.30	4.82	12.86	13.72	2.20	NA	1.27	2.18	5.07	1.09	3.76	11.48
8	6.37	4.48	9.42	15.43	5.88	9.80	1.89	3.01	3.60	1.48	1.85	1.46	1.27	1.96	1.55
9	3.75	2.57	6.27	14.66	11.93	13.58	1.58	1.12	3.28	2.79	9.47	6.31	2.85	9.27	4.87
10	NA	NA	5.65	NA	NA	17.55	NA	NA	24.81	NA	NA	1.09	NA	NA	NA
11	4.58	3.00	8.06	6.08	6.17	8.74	1.52	1.71	6.76	1.49	1.37	1.05	1.12	1.71	1.20
12	16.11	4.82	9.59	12.26	14.27	12.11	28.86	11.74	NA	2.17	5.17	6.50	NA	NA	NA
13	4.78	2.90	124.63	9.52	10.31	38.95	17.50	3.06	NA	1.27	1.56	65.08	1.56	2.93	71.73
14	18.56	20.81	12.65	12.98	12.73	6.03	28.74	28.93	3.54	8.59	9.35	1.55	7.78	9.08	1.24
15	7.06	3.52	3.83	9.22	7.35	12.67	1.75	1.67	22.10	2.07	1.56	9.81	1.03	1.88	2.88
16	3.50	2.59	121.34	9.81	11.96	15.60	3.26	2.02	NA	1.24	1.32	14.67	1.62	2.21	10.00
17	4.58	3.42	5.10	22.05	17.49	17.98	1.89	1.96	21.37	1.41	1.05	1.15	1.07	2.27	0.93
18	4.47	3.79	10.55	12.39	5.57	NA	1.92	1.96	6.17	1.62	1.59	1.78	1.84	2.71	2.10
19	3.76	3.52	8.09	8.83	4.88	13.80	2.28	1.77	3.98	1.18	1.86	1.84	1.01	1.98	1.06
20	5.52	2.23	5.53	12.06	9.82	16.20	10.29	1.75	3.35	19.42	10.80	12.09	1.62	3.47	1.84
21	4.05	3.26	5.97	23.23	8.79	6.97	1.40	2.01	3.56	4.44	1.13	1.24	2.78	1.14	1.21
22	7.07	5.77	125.06	12.03	5.98	101.68	19.25	6.34	NA	1.86	1.49	97.61	1.83	1.52	NA
23	3.63	4.50	NA	17.17	6.48	89.74	12.58	4.59	NA	1.63	1.22	8.98	1.24	1.27	19.40
24	2.67	2.31	129.92	12.50	13.22	98.24	2.91	1.85	NA	2.41	1.25	48.50	1.78	1.24	62.42
25	8.39	3.10	NA	9.57	14.96	NA	4.79	1.80	NA	2.29	6.89	3.33	3.51	5.77	8.87
26	13.38	NA	125.54	13.98	NA	31.64	12.58	NA	NA	1.26	NA	81.29	NA	NA	NA
27	10.74	3.85	6.13	14.20	10.74	21.68	25.99	2.92	16.31	1.32	2.23	1.44	1.19	2.65	1.42
28	19.94	30.00	11.23	14.65	9.71	13.75	6.44	9.48	4.98	22.17	13.28	16.76	46.41	20.87	33.98
29	9.47	7.62	8.24	3.99	3.77	6.19	2.41	2.26	6.38	2.74	1.32	1.68	1.51	1.46	1.40
30	6.32	5.35	10.17	7.12	6.85	12.98	2.72	1.35	2.55	2.48	1.25	1.20	1.65	1.31	1.12
31	9.73	3.88	8.66	11.95	9.59	12.56	1.53	1.56	2.64	1.30	2.74	1.72	1.37	3.49	1.47
32	11.92	2.34	3.90	10.21	12.81	14.81	40.74	1.32	4.63	5.05	1.52	1.63	4.28	1.01	2.30
33	5.24	2.13	155.40	9.99	8.57	NA	9.63	1.80	NA	1.59	1.90	182.68	1.36	3.19	NA
34	10.08	3.71	5.14	19.47	17.28	20.76	1.33	1.60	13.03	3.75	8.43	9.06	3.73	10.10	6.98
35	21.38	4.71	9.18	10.07	16.06	14.58	33.94	2.74	NA	12.58	12.61	13.13	1.20	2.34	1.56
36	7.61	10.59	15.00	10.19	7.16	9.70	6.73	13.54	15.27	1.25	4.06	2.75	1.79	5.05	3.50
37	26.08	4.56	50.09	22.86	14.06	18.82	1.57	1.69	23.42	2.50	1.34	1.75	1.84	1.22	4.27
38	5.99	2.16	5.29	7.72	5.56	17.91	11.12	2.91	12.56	1.78	1.51	3.93	1.15	1.67	3.67
39	3.56	2.48	5.13	12.09	9.77	15.45	1.38	2.10	1.43	1.18	1.27	1.75	1.24	2.97	1.44
40	11.15	5.74	120.90	20.91	13.28	NA	1.73	5.68	115.96	2.30	6.05	61.26	3.32	5.17	54.08
41	25.52	2.82	141.90	18.22	13.93	NA	21.89	2.60	139.11	1.44	1.32	51.76	1.09	1.99	55.34
42	9.28	2.74	2.97	5.03	13.79	25.84	11.52	3.13	17.80	1.12	2.34	2.62	1.41	2.48	2.73
43	7.21	4.15	4.74	18.28	10.47	NA	6.19	2.71	19.28	4.01	1.20	1.59	1.86	1.13	1.82
44	20.87	2.15	4.57	17.76	11.41	15.65	7.06	1.66	2.55	2.61	1.54	1.05	1.09	1.85	1.03
45	17.32	2.62	6.89	7.72	11.57	12.33	7.96	1.41	6.36	1.37	1.93	1.06	1.08	2.20	0.99
46	6.46	2.83	6.64	23.27	16.99	22.37	2.74	2.46	8.00	2.31	6.15	5.54	4.16	8.46	7.31
47	8.16	2.59	4.95	7.85	8.29	12.50	1.64	1.97	2.29	1.13	2.25	1.96	1.21	2.96	1.56
48	8.47	3.23	7.36	22.03	13.20	27.41	10.35	1.84	11.64	1.65	2.06	1.37	1.39	2.21	1.13
49	15.13	38.52	8.58	21.28	25.93	27.43	14.37	12.74	8.02	13.27	13.20	13.72	6.82	16.65	12.81
50	14.12	4.54	6.39	14.14	8.21	23.55	4.09	1.86	11.21	2.33	1.19	2.62	1.19	1.04	2.82
51	7.25	9.63	11.90	6.46	8.70	10.50	1.97	1.26	1.39	1.48	1.63	1.51	1.20	2.98	1.31
52	19.44	2.67	4.45	21.31	9.82	NA	5.92	1.89	9.25	2.10	7.54	5.06	4.07	11.19	6.61
53	3.64	2.20	3.90	15.55	13.99	18.93	1.28	1.09	1.41	2.72	7.25	8.46	3.57	8.32	7.27
54	29.69	3.15	3.99	17.76	7.32	12.42	41.04	2.59	25.77	1.11	2.37	2.11	1.14	2.36	3.67
55	43.45	4.04	5.09	7.53	6.22	8.19	8.79	1.25	3.28	2.60	0.86	0.94	1.18	1.51	0.82
56	5.64	4.83	6.90	10.32	9.01	16.28	1.76	1.62	1.66	2.58	1.75	1.22	1.48	2.94	1.20
57	NA	NA	NA	10.62	6.02	10.33	1.66	1.75	6.76	1.19	1.19	1.79	1.33	2.14	2.88
58	NA	NA	4.95	NA	NA	11.59	NA	NA	27.47	NA	NA	1.08	NA	NA	1.17
59	7.63	2.72	4.50	11.21	8.54	15.50	1.33	1.63	2.05	1.43	4.90	4.76	2.30	5.70	4.66
60	3.25	2.18	5.23	23.87	16.42	16.84	1.55	1.84	3.89	1.75	2.52	1.52	1.29	3.92	1.46
61	4.05	3.22	NA	9.92	6.71	8.41	1.50	1.95	NA	1.46	1.50	2.29	1.29	1.35	1.83
62	2.49	1.86	3.77	10.06	12.21	18.52	8.11	2.02	16.82	1.54	1.66	1.00	1.15	2.17	1.10
63	5.59	3.43	5.34	10.98	11.49	12.58	19.80	2.96	13.59	1.19	1.92	1.83	1.45	3.28	2.26
64	3.03	3.02	6.05	11.38	9.11	12.77	1.87	1.62	2.70	1.94	1.82	1.49	1.14	2.94	1.33
65	18.41	4.42	18.88	15.80	8.72	15.85	14.92	1.26	8.68	3.13	0.98	1.90	2.15	1.43	2.18
66	6.02	2.54	4.98	11.78	10.35	12.23	16.54	1.56	16.16	1.73	1.48	1.28	1.93	1.55	1.40
67	10.19	2.36	4.38	8.53	10.18	15.69	6.89	2.03	9.30	1.27	1.46	1.81	1.28	1.64	1.42
68	3.22	3.59	5.54	11.70	8.39	11.54	8.58	1.91	15.14	1.31	1.27	2.13	1.54	3.36	2.20
69	11.53	7.87	11.41	7.14	16.01	17.44	6.33	3.96	9.67	3.63	4.79	6.99	3.43	5.46	5.01
70	14.93	26.40	4.74	17.92	8.26	12.89	15.19	102.80	17.67	13.53	9.66	12.07	6.77	7.25	8.24
71	3.27	6.25	13.03	9.32	10.82	10.71	30.15	2.33	4.10	7.65	6.34	5.70	1.33	2.04	1.25
72	3.38	2.28	3.90	17.30	5.23	21.30	7.87	2.10	17.83	2.23	1.47	1.36	1.69	2.46	1.57
73	23.41	6.75	11.29	12.05	8.63	NA	7.36	4.16	16.93	1.23	3.53	2.38	1.21	2.65	1.73
74	6.45	8.67	6.24	18.74	14.90	19.06	17.62	51.50	24.59	15.67	14.91	18.54	9.12	7.01	13.02
75	3.53	3.41	5.44	10.74	7.19	10.95	5.81	2.59	6.37	1.42	1.21	3.15	1.20	1.97	1.87
76	7.03	3.41	4.97	9.31	7.83	18.27	2.35	2.34	18.01	1.16	1.38	2.37	1.23	1.21	1.44
77	7.19	3.12	4.63	7.07	9.19	14.34	6.51	2.17	18.88	1.04	1.61	1.40	1.08	1.56	1.62
78	7.18	2.44	131.84	19.76	8.16	12.23	17.79	2.20	133.97	2.01	2.00	80.90	1.45	2.09	68.18
79	16.13	2.85	7.67	14.01	6.15	18.81	24.63	1.90	28.78	1.85	1.28	1.35	1.01	1.28	1.14
80	4.91	3.02	5.97	18.21	14.45	18.21	15.73	2.40	13.23	1.00	1.49	1.06	0.75	1.39	0.68
81	4.17	4.14	8.00	13.42	14.91	15.48	5.08	3.69	4.33	1.90	1.72	1.98	1.56	3.27	2.19
82	16.11	3.90	4.57	8.80	13.07	15.08	17.91	1.44	1.20	1.15	1.84	1.00	0.97	2.13	1.02
83	3.47	2.49	5.47	8.43	10.38	10.81	1.39	1.83	2.32	1.83	1.17	1.11	1.25	1.54	1.24
84	5.79	2.68	4.92	5.93	5.12	10.78	3.05	1.64	5.17	1.22	3.29	3.41	1.26	3.50	1.80
85	4.30	3.90	118.66	10.21	8.86	116.65	1.33	2.39	NA	1.62	3.50	57.09	2.78	3.64	48.61
86	2.82	2.15	4.60	8.57	9.20	10.82	1.45	1.59	1.91	1.71	5.57	4.95	2.17	6.05	3.61
87	14.44	2.37	132.03	7.59	8.23	6.95	6.43	1.54	NA	1.81	1.35	2.00	1.39	1.58	5.79
88	4.71	4.30	10.46	10.83	6.63	9.98	24.87	2.31	18.84	3.13	6.92	5.13	3.11	7.69	3.71
89	6.47	4.36	9.06	12.27	11.00	14.99	8.83	1.67	8.82	2.29	5.88	3.91	3.14	7.60	4.18
90	9.12	4.17	6.19	4.43	6.96	8.83	1.84	2.63	7.78	1.45	1.38	2.24	1.23	3.72	2.55
91	2.87	2.91	3.96	11.43	9.04	14.31	1.71	1.57	8.83	1.53	5.23	2.42	1.60	6.57	2.17
92	3.43	4.62	119.76	10.95	9.16	NA	5.73	6.54	125.29	1.35	1.15	65.07	1.15	1.21	58.27
93	2.21	5.41	11.40	8.60	4.43	NA	3.60	9.44	16.95	2.23	1.40	1.51	1.28	1.13	1.49
94	17.54	7.97	9.01	11.53	4.87	11.75	15.01	4.02	10.68	1.43	1.95	1.45	1.25	2.29	1.33
95	13.60	5.63	105.68	8.48	13.23	NA	5.30	2.43	NA	3.77	2.11	61.07	1.68	5.05	66.44
96	4.09	3.84	5.60	18.17	9.09	21.38	2.70	1.86	17.97	1.73	1.57	1.18	1.23	1.98	1.03
97	4.28	2.46	5.10	15.21	13.76	17.11	1.45	1.71	1.27	1.58	2.30	3.91	1.67	3.54	2.91
98	5.22	2.42	NA	9.86	5.44	8.07	4.58	2.10	NA	1.42	1.42	13.42	1.21	1.72	11.70
99	5.80	4.15	5.58	10.10	13.85	20.66	15.47	2.92	13.79	1.06	2.02	1.91	0.97	1.96	0.99
100	3.13	2.78	2.95	8.36	9.60	10.88	1.46	2.00	4.24	2.08	1.21	1.03	1.17	1.44	1.02
101	15.48	3.18	5.67	NA	NA	NA	1.61	1.46	5.35	1.25	1.99	2.00	1.27	3.07	1.43
102	3.18	3.32	4.87	5.61	5.21	13.43	8.02	2.82	4.91	1.56	3.89	2.65	2.05	5.09	3.43
103	11.04	3.11	12.71	16.72	5.27	10.22	27.26	2.50	NA	12.80	10.04	14.16	1.48	1.96	2.72
104	12.04	4.30	4.43	11.73	6.33	14.61	22.60	5.68	29.58	0.96	1.62	1.73	NA	NA	NA
105	8.40	3.59	4.01	16.79	5.45	18.11	3.81	2.31	8.54	1.40	1.58	0.87	1.19	1.70	1.06
106	5.69	2.47	3.23	10.73	7.73	9.73	14.35	1.65	3.56	1.28	1.19	1.08	1.26	1.57	1.13
107	11.58	3.13	6.88	11.01	5.66	14.48	2.01	1.72	1.70	1.28	1.17	1.05	1.05	1.68	0.90
108	8.27	6.64	8.06	14.25	10.78	12.16	5.91	2.78	4.08	11.98	18.39	13.52	3.71	5.67	5.97
109	2.15	2.12	3.96	2.04	5.02	5.77	1.68	1.66	1.89	1.24	1.80	1.93	1.21	2.14	1.26
110	2.67	164.41	3.18	5.99	32.85	5.55	1.35	11.86	1.89	0.98	32.46	2.13	1.21	32.72	2.13
111	3.08	3.08	6.30	NA	NA	NA	1.58	1.89	3.14	1.66	1.45	1.14	1.52	1.82	1.11
112	2.18	1.83	4.51	5.78	12.28	9.76	1.35	1.38	2.54	2.98	1.11	1.29	1.26	1.65	1.01
113	6.28	3.60	5.60	9.88	4.67	10.40	1.99	1.60	4.32	2.42	1.17	1.11	1.48	2.39	1.11
114	3.09	2.45	3.20	3.88	4.04	4.44	5.09	1.32	3.09	1.61	1.55	1.21	1.00	1.70	1.08
115	7.12	2.69	6.07	NA	NA	NA	27.12	2.84	9.57	NA	NA	NA	NA	NA	NA
116	5.18	2.48	5.29	6.72	9.03	14.11	1.40	1.54	6.25	1.76	2.13	1.29	1.33	2.92	1.30
117	3.02	2.91	2.37	3.99	2.88	5.33	2.42	2.74	2.58	1.42	4.09	3.49	1.72	4.49	2.57
118	4.93	2.16	3.72	12.99	12.40	NA	1.31	1.42	2.83	1.89	0.96	0.96	1.07	2.31	0.95
119	4.74	1.96	6.99	14.90	5.48	NA	23.46	1.30	2.95	1.50	1.23	1.33	1.38	1.53	1.97
120	4.84	2.41	2.59	8.48	6.73	11.63	1.54	1.42	1.77	1.51	4.93	3.86	2.14	4.68	2.81
121	5.60	3.49	6.39	8.40	1.99	5.29	1.45	1.70	1.76	1.46	2.16	1.50	1.13	2.62	1.75
122	4.23	2.97	3.47	12.21	8.94	12.91	1.31	1.67	1.06	1.87	3.06	4.93	2.71	3.20	4.66
123	9.05	2.20	4.24	NA	NA	NA	1.35	1.14	5.01	1.15	2.06	1.27	8.27	2.28	1.04
124	4.79	2.57	8.38	22.13	12.88	15.94	1.98	1.46	8.19	1.99	1.82	1.48	1.59	2.26	1.96
125	3.34	2.02	5.39	13.15	2.51	5.43	2.25	1.45	6.15	2.03	2.10	1.10	1.52	2.42	1.26
126	3.52	1.73	5.85	6.56	2.62	NA	1.78	1.39	7.23	1.63	5.13	3.29	2.15	7.06	3.44
127	23.56	5.57	6.22	NA	NA	NA	9.51	4.98	6.05	1.52	4.66	5.01	2.76	5.74	5.18
128	13.92	9.36	6.85	NA	NA	NA	2.64	3.53	3.87	2.05	1.77	1.83	3.96	7.85	5.37
129	2.19	2.28	3.80	NA	NA	NA	7.22	1.40	1.52	1.07	2.89	2.73	1.23	2.83	1.92
130	4.28	2.97	4.02	5.09	2.64	5.93	1.69	1.26	13.32	1.73	2.44	2.08	1.44	2.80	1.46
131	4.80	3.76	7.54	15.98	5.50	13.47	3.28	3.90	3.33	1.96	5.04	4.74	2.45	5.02	3.79

Supplementary Table 5. Detailed list of structure comparisons per individual for prostate, rectum, bladder, and bilateral femoral heads (femur_L, femur_R). Comparisons all versus clinician-delineated contours via 95% Hausdorff distance (HD) in millimeters. Results are for model-based (MB), deep learning (DL), and atlas-based (AB) algorithms.

ID	Prostate			Rectum			Bladder			Femur_L			Femur_R		
	MB	DL	AB	MB	DL	AB	MB	DL	AB	MB	DL	AB	MB	DL	AB
1	38.81	5.21	10.68	58.16	34.08	40.22	44.79	3.10	4.73	38.03	3.12	2.29	37.12	4.02	3.38
2	33.01	6.58	9.21	46.98	30.56	NA	28.32	3.96	51.45	12.02	2.69	4.03	3.26	3.17	3.85
3	14.60	6.50	11.00	19.54	42.02	48.09	16.65	6.78	40.12	8.01	2.20	2.11	2.49	4.08	1.92
4	7.94	5.83	24.50	34.15	30.15	28.03	5.26	3.29	18.67	3.43	4.24	4.33	2.54	8.06	2.72
5	14.53	8.34	80.83	43.77	24.46	28.32	7.62	4.14	NA	4.28	20.19	28.06	4.53	26.14	40.26
6	31.82	24.49	25.01	32.00	55.66	58.56	44.65	7.05	24.53	16.00	2.31	2.71	6.08	3.05	3.89
7	35.76	11.03	NA	16.95	21.88	42.01	38.93	6.80	NA	2.62	12.03	24.00	2.26	26.31	35.77
8	14.27	11.22	23.18	51.18	23.88	31.93	4.92	10.00	12.52	4.08	7.07	4.15	2.49	8.19	4.42
9	8.66	6.00	15.39	40.15	37.33	40.58	2.80	2.29	15.73	16.99	51.59	31.18	18.26	51.89	26.36
10	NA	NA	15.62	NA	NA	48.65	NA	NA	60.14	NA	NA	3.11	NA	NA	NA
11	13.45	9.83	20.72	25.76	32.10	28.13	3.97	3.95	22.94	4.39	2.95	2.36	2.18	4.76	2.91
12	35.87	10.19	21.62	46.97	39.36	36.60	63.76	45.78	NA	10.06	31.62	36.51	NA	NA	NA
13	14.40	6.48	144.31	35.70	38.16	87.25	54.19	9.05	NA	2.51	4.08	94.95	3.46	16.00	108.07
14	42.84	48.08	29.87	43.91	34.76	16.84	53.37	53.55	16.13	19.60	21.23	6.20	18.06	24.25	3.02
15	22.35	13.63	12.34	36.20	35.18	44.07	5.47	3.14	85.98	11.18	6.05	38.24	2.23	8.17	12.07
16	8.81	5.49	142.23	25.17	38.28	50.16	12.66	4.99	NA	2.50	2.77	50.35	3.55	10.26	34.38
17	12.75	8.65	11.37	66.58	53.12	52.46	6.09	4.12	59.20	4.32	2.50	2.90	2.27	12.11	2.14
18	11.67	8.04	24.91	43.70	25.44	NA	3.85	4.86	22.40	4.22	3.87	6.18	5.42	15.02	6.08
19	10.23	8.86	18.52	36.00	24.26	43.89	6.99	3.66	14.03	2.38	7.03	8.61	2.21	8.06	2.52
20	16.70	5.68	15.15	45.57	36.18	54.32	29.47	3.69	10.01	96.01	63.53	55.70	4.58	21.41	9.25
21	11.13	8.58	11.79	71.14	34.07	18.36	3.14	4.31	16.05	28.44	2.53	2.75	18.11	2.47	2.54
22	17.00	21.25	150.01	38.29	24.25	132.56	54.16	20.52	NA	5.07	3.59	144.87	4.95	3.65	NA
23	11.28	14.98	NA	57.14	26.18	114.20	46.83	22.22	NA	6.08	2.88	26.27	2.87	2.68	50.68
24	6.58	5.61	148.35	37.28	40.63	118.14	10.01	4.99	NA	13.76	3.05	85.99	6.15	2.81	104.29
25	23.83	6.78	NA	31.95	66.44	NA	24.93	4.03	NA	13.00	42.37	11.44	24.04	34.83	26.79
26	22.37	NA	152.00	39.55	NA	70.40	33.53	NA	NA	2.44	NA	112.40	NA	NA	NA
27	26.05	8.39	12.33	48.01	46.40	66.03	58.39	6.24	34.57	2.82	12.06	4.35	2.33	16.24	4.47
28	38.16	46.23	27.18	55.82	36.95	38.06	25.42	31.22	21.34	73.30	50.04	58.85	119.41	66.48	108.29
29	28.85	21.74	19.71	17.90	18.01	21.01	7.49	6.09	27.18	12.78	3.10	4.70	3.54	3.34	3.66
30	14.60	14.76	21.68	34.05	29.55	44.40	10.83	2.70	8.77	14.01	2.84	2.56	5.26	2.89	2.62
31	22.35	8.66	15.70	38.06	36.14	47.52	3.01	3.11	12.06	2.46	16.35	7.95	3.01	23.15	4.01
32	30.62	5.85	9.39	30.44	41.51	47.31	86.07	2.65	17.33	32.57	4.76	4.46	28.50	2.29	10.08
33	13.33	4.24	176.53	29.09	35.24	NA	28.79	3.62	NA	4.06	6.13	224.35	2.66	18.10	NA
34	24.23	9.73	13.68	58.79	52.54	60.91	3.52	3.81	47.81	22.04	45.82	40.32	23.31	56.02	34.00
35	51.75	15.92	27.36	38.56	40.22	38.59	75.70	6.08	NA	33.34	49.95	36.97	2.30	12.67	4.41
36	25.03	39.07	40.03	38.41	30.00	34.52	29.76	48.64	44.09	2.59	27.35	16.95	6.38	33.26	22.16
37	44.74	10.28	70.66	75.89	47.77	53.66	3.52	3.09	58.87	13.01	2.90	6.41	7.01	2.77	18.41
38	22.27	4.96	10.40	18.23	23.13	57.35	32.75	8.53	34.25	6.20	3.43	15.82	2.29	3.22	14.59
39	8.39	6.50	10.24	46.68	37.37	50.54	2.81	7.83	3.50	2.29	2.70	8.44	2.41	19.01	4.42
40	28.78	16.05	143.73	51.24	44.16	NA	5.54	22.92	134.12	12.99	35.09	111.82	22.18	30.17	97.13
41	62.41	8.45	160.08	64.87	52.91	NA	61.47	5.40	154.70	3.33	2.97	85.12	2.23	8.42	92.56
42	27.07	7.93	6.11	18.52	46.50	66.96	49.12	9.39	46.16	2.31	12.21	15.58	3.12	12.23	16.03
43	19.53	14.40	11.79	42.39	34.76	NA	18.20	8.01	58.56	24.22	2.66	4.54	6.24	2.25	4.81
44	41.96	6.38	8.53	46.03	42.54	51.05	21.86	3.20	9.18	14.29	3.67	2.56	2.51	6.21	2.72
45	34.50	7.41	12.53	32.35	40.99	41.83	30.56	3.06	29.39	2.98	7.55	2.43	2.20	12.01	2.28
46	17.99	5.63	14.13	58.28	45.76	54.87	11.42	7.39	28.31	10.10	36.13	28.36	26.01	44.97	34.27
47	22.36	6.06	13.78	29.09	38.30	48.59	4.29	5.18	9.47	2.24	12.95	10.12	2.29	20.01	6.05
48	25.53	7.02	13.29	67.93	47.30	70.04	32.71	3.14	32.28	6.38	10.07	3.28	3.31	10.37	2.42
49	29.35	55.99	21.10	54.67	67.01	72.01	41.99	41.40	23.20	36.38	48.26	41.02	30.19	44.02	36.95
50	31.71	9.93	15.60	38.55	33.24	64.43	16.72	5.02	36.97	10.13	3.12	11.19	3.11	2.83	12.87
51	31.65	36.33	39.73	25.80	33.96	33.55	6.74	3.04	4.39	3.57	4.12	3.67	2.86	18.41	3.19
52	35.97	6.45	9.27	60.06	36.25	NA	23.90	4.55	28.51	9.35	45.14	26.23	26.05	58.10	32.25
53	10.00	5.10	9.66	48.34	44.03	52.58	3.74	2.43	6.74	15.27	42.34	40.01	22.48	48.00	35.01
54	56.99	6.59	9.01	43.60	31.44	41.96	89.73	7.40	71.08	2.26	14.03	10.32	2.32	13.19	21.19
55	66.29	11.74	12.58	29.89	40.07	26.30	35.65	2.38	10.14	16.11	2.11	2.78	4.09	3.46	2.27
56	19.28	13.16	15.04	46.01	39.03	54.01	4.71	4.05	5.53	12.50	5.49	2.60	3.19	18.31	2.90
57	NA	NA	NA	40.47	30.36	34.18	4.57	4.47	21.15	2.35	2.59	7.09	2.48	10.65	14.08
58	NA	NA	10.06	NA	NA	43.41	NA	NA	70.04	NA	NA	2.51	NA	NA	2.61
59	17.02	8.72	10.68	36.24	34.14	50.36	2.60	2.88	6.41	2.93	30.20	28.02	14.01	35.94	28.01
60	8.43	5.91	14.69	65.24	48.22	46.75	3.20	4.15	11.74	6.19	14.18	3.66	2.38	26.14	4.10
61	10.72	7.41	NA	36.37	27.07	30.02	3.07	5.95	NA	4.74	4.11	8.60	3.39	4.03	6.42
62	6.73	4.20	8.08	32.32	43.54	50.01	29.42	4.01	44.32	6.02	6.03	2.17	2.60	12.13	2.53
63	12.74	10.81	13.64	40.99	44.01	44.27	47.57	10.09	40.96	2.34	8.11	8.00	3.01	20.46	10.89
64	7.69	7.10	10.22	47.17	36.06	47.18	4.35	3.42	9.64	8.91	6.38	6.02	2.29	20.06	4.29
65	47.23	12.29	31.52	52.00	38.39	49.10	51.97	3.01	24.97	19.78	2.29	7.49	11.61	2.82	8.92
66	16.41	5.92	10.61	38.02	38.00	38.97	49.47	3.28	42.16	4.47	2.96	3.37	6.15	3.16	3.82
67	22.07	5.45	8.83	26.56	36.49	50.10	24.11	5.32	28.16	2.57	3.46	8.30	2.51	4.06	4.23
68	9.75	8.69	12.66	42.15	35.70	43.19	38.06	4.36	36.92	2.86	3.06	9.05	3.70	22.93	8.53
69	30.17	29.03	35.12	18.56	45.33	55.69	17.81	15.36	30.29	20.12	28.03	34.02	20.06	32.01	26.46
70	27.64	41.81	10.27	59.33	30.03	39.02	34.94	155.66	40.40	35.67	38.49	32.45	24.05	24.01	25.38
71	8.72	18.65	28.58	34.25	30.88	33.11	76.86	7.94	13.80	27.04	27.77	20.19	2.75	4.31	3.21
72	10.60	5.15	7.89	62.49	21.62	65.31	34.26	4.78	43.58	9.17	4.05	3.72	4.15	9.17	5.26
73	46.01	18.25	26.96	44.87	32.69	NA	29.90	20.03	51.41	2.41	24.03	13.78	2.36	16.10	8.50
74	14.04	18.33	16.02	58.93	55.63	56.35	46.19	79.60	62.49	58.19	61.08	56.61	34.73	37.97	49.31
75	9.75	10.42	13.39	40.83	30.07	38.06	23.33	9.63	20.80	4.02	2.82	21.49	2.40	8.24	8.06
76	16.05	8.99	10.28	32.41	36.04	56.01	6.16	7.03	47.83	2.42	3.00	12.55	2.63	2.85	4.31
77	14.13	7.03	9.64	28.47	42.91	48.00	29.68	4.15	55.92	2.12	6.03	5.10	2.16	4.25	6.26
78	16.20	5.57	150.19	62.34	36.00	42.06	57.15	5.87	150.69	12.06	10.00	121.35	6.05	8.88	112.21
79	30.98	6.59	13.08	42.00	27.09	54.57	66.45	3.76	76.43	7.41	2.77	2.92	2.12	2.80	2.50
80	11.73	6.26	11.10	62.46	52.33	54.28	49.08	6.07	46.52	2.28	4.00	3.30	1.73	3.30	1.91
81	9.88	11.52	15.82	52.03	52.70	51.22	19.07	15.39	14.81	6.29	6.04	9.14	3.55	21.20	9.49
82	32.25	7.77	9.34	40.03	42.66	50.62	46.54	2.95	3.01	2.56	8.19	2.69	2.04	10.22	2.43
83	8.33	5.62	9.80	30.16	41.55	39.95	2.99	3.66	9.47	6.64	2.52	2.33	2.58	3.68	2.50
84	20.19	8.86	12.12	24.00	23.09	39.82	14.85	3.06	27.07	2.41	21.08	20.23	2.36	24.00	9.31
85	9.19	10.15	141.31	38.32	35.06	146.22	3.39	6.76	NA	3.82	17.15	89.79	15.06	16.07	83.15
86	7.73	5.22	10.30	36.06	40.73	40.28	2.68	3.89	7.54	4.31	34.45	28.05	10.33	38.00	22.02
87	28.59	4.79	152.08	27.92	33.59	26.99	26.55	3.25	NA	10.18	3.01	9.22	6.02	4.00	23.47
88	12.10	13.96	25.72	44.56	29.26	37.99	72.32	7.13	59.83	15.60	38.74	25.33	16.97	45.57	17.87
89	22.04	11.92	28.65	38.94	36.78	44.00	29.00	2.87	27.78	12.01	37.09	20.76	20.25	46.00	22.74
90	22.74	9.18	12.21	19.93	30.27	34.35	4.00	5.56	31.20	4.11	3.51	7.51	2.55	25.43	9.83
91	7.04	6.83	8.03	38.23	36.01	46.38	4.66	3.26	35.80	2.91	34.77	12.86	3.54	44.19	12.13
92	9.45	13.03	143.89	37.47	34.00	NA	25.57	24.28	138.58	4.22	2.44	111.62	2.46	3.20	100.24
93	4.50	18.57	29.62	34.43	22.95	NA	15.13	35.19	52.01	12.10	3.23	4.14	2.79	2.54	4.27
94	40.87	20.12	18.18	37.48	23.36	42.33	46.58	15.13	31.18	4.01	8.17	4.49	2.63	14.01	3.62
95	28.45	14.07	129.37	26.30	48.42	NA	15.54	6.04	NA	20.05	6.99	114.44	6.21	34.12	118.05
96	11.24	10.13	15.94	62.25	38.30	58.27	9.41	4.18	41.66	7.55	4.47	3.48	2.45	10.01	2.15
97	9.76	5.64	9.60	52.53	49.93	56.11	3.76	3.79	2.80	3.23	12.10	22.15	3.74	22.11	16.20
98	15.96	5.49	NA	33.47	24.10	29.56	16.55	5.00	NA	4.02	3.44	39.69	2.66	6.02	42.02
99	16.77	10.69	12.24	30.25	52.19	60.20	37.05	10.51	52.02	2.26	10.66	10.36	2.09	10.01	2.42
100	7.52	5.20	6.04	28.04	36.03	38.01	3.61	4.55	19.61	10.01	2.51	2.19	2.35	3.32	2.40
101	42.10	9.08	12.36	NA	NA	NA	3.73	2.80	23.07	2.51	8.26	10.07	2.44	20.17	4.15
102	9.15	8.81	10.59	13.47	24.84	42.12	29.00	6.63	18.03	3.06	25.11	16.03	8.30	32.37	20.27
103	23.27	8.02	32.66	62.02	26.05	38.00	58.88	6.97	NA	30.61	44.35	38.91	4.21	4.22	10.79
104	29.74	10.61	9.49	46.58	36.03	58.04	56.38	21.23	75.80	2.11	4.64	7.35	NA	NA	NA
105	25.42	9.31	9.81	52.51	22.04	48.06	15.38	6.33	24.55	4.25	6.01	2.06	2.71	6.04	2.34
106	16.63	6.10	8.64	39.71	36.97	38.16	49.26	3.86	13.53	2.92	2.59	2.35	2.86	4.03	2.50
107	23.00	6.81	12.35	39.19	28.37	53.62	6.22	4.37	5.01	3.65	3.02	3.07	2.91	6.00	2.54
108	16.38	20.23	22.16	39.60	32.53	36.02	19.55	7.98	11.24	45.34	50.70	38.24	23.07	32.46	32.01
109	6.16	4.97	7.50	8.23	28.91	19.72	4.69	3.96	6.81	2.54	8.06	8.13	2.37	10.08	2.65
110	7.38	188.18	8.16	17.74	74.41	28.02	2.43	38.69	8.94	1.81	103.03	13.02	2.28	93.34	13.08
111	8.00	6.40	12.17	NA	NA	NA	3.93	3.92	13.00	6.03	3.27	2.44	4.18	6.01	2.38
112	6.14	4.19	11.57	26.43	45.57	34.24	3.03	2.61	10.35	16.51	2.35	3.03	2.67	4.15	2.33
113	15.86	10.99	12.80	34.02	21.73	38.64	5.59	4.16	15.44	12.13	2.57	2.32	3.38	12.60	2.60
114	8.97	7.03	7.23	10.80	24.03	22.22	23.15	2.76	17.50	6.06	4.11	2.50	1.79	6.03	2.27
115	20.47	6.75	16.28	NA	NA	NA	80.20	10.24	32.46	NA	NA	NA	NA	NA	NA
116	19.61	6.66	13.22	27.17	45.39	63.93	2.77	3.01	25.28	4.73	8.04	2.56	2.45	16.11	2.50
117	6.07	5.65	5.38	14.13	14.13	24.91	4.98	5.90	5.58	3.81	27.34	22.01	6.34	31.10	16.24
118	10.39	4.73	6.59	48.01	40.02	NA	2.45	2.80	10.11	9.31	1.91	2.03	2.08	15.02	1.82
119	14.64	4.88	17.80	38.32	24.28	NA	71.88	2.57	9.88	5.28	2.94	4.17	3.49	3.47	6.72
120	16.34	6.48	6.19	30.02	27.15	40.38	3.10	3.05	5.15	2.52	30.14	22.48	6.28	29.19	15.10
121	11.52	6.16	12.13	25.64	5.62	14.43	3.44	3.83	5.14	3.50	11.01	3.13	2.13	16.46	6.77
122	9.38	7.76	8.32	39.35	33.64	39.42	3.48	3.94	3.82	4.03	18.01	27.06	13.20	18.03	24.64
123	24.91	5.22	11.89	NA	NA	NA	2.71	2.80	23.89	2.41	10.02	4.11	33.29	10.37	2.12
124	11.83	6.59	17.74	67.90	64.04	52.00	4.46	4.02	24.80	9.30	6.09	4.62	4.35	12.06	6.64
125	8.12	4.60	9.91	55.74	10.76	17.49	8.45	2.68	16.28	9.29	10.21	2.44	3.17	14.02	2.60
126	11.23	4.38	16.44	20.10	10.93	NA	7.39	2.39	31.03	4.03	32.01	18.94	8.10	43.95	19.24
127	52.06	20.32	19.46	NA	NA	NA	47.83	24.46	30.20	3.24	28.19	28.11	15.86	33.42	28.12
128	29.92	38.61	32.62	NA	NA	NA	12.11	15.78	16.08	5.54	3.54	4.46	14.48	25.48	20.68
129	6.04	5.08	8.75	NA	NA	NA	33.76	2.84	4.29	1.97	18.16	17.05	2.30	17.15	11.34
130	12.42	6.64	8.84	17.44	11.87	32.46	3.67	2.43	46.32	4.52	11.09	9.52	2.74	15.35	3.11
131	13.10	9.70	16.83	50.00	24.17	27.82	12.56	11.57	12.19	4.61	30.22	24.48	10.16	30.28	20.08
